# *Ntoco* Promotes Ferroptosis via Hnrnpab‐Mediated NF‐κB/Lcn2 Axis Following Traumatic Brain Injury in Mice

**DOI:** 10.1111/cns.70282

**Published:** 2025-02-20

**Authors:** Qiang Wang, Hanjun Zuo, Huaqin Sun, Xiao Xiao, Zhao Wang, Tingyu Li, Xiaolei Luo, Yanyun Wang, Tao Wang, Juanjuan Li, Linbo Gao

**Affiliations:** ^1^ Laboratory of Molecular Translational Medicine, Center for Translational Medicine, Key Laboratory of Birth Defects and Related Diseases of Women and Children (Sichuan University), Ministry of Education, West China Second University Hospital Sichuan University Chengdu Sichuan China; ^2^ Department of Anatomy and Histology & Embryology, Faculty of Basic Medical Sciences Kunming Medical University Kunming Yunnan China; ^3^ SCU‐CUHK Joint Laboratory for Reproductive Medicine, Key Laboratory of Birth Defects and Related Diseases of Women and Children (Sichuan University), Ministry of Education, Department of Pediatrics West China Second University Hospital, Sichuan University Chengdu China

**Keywords:** ferroptosis, long non‐coding RNAs, mechanism, traumatic brain injury, ubiquitination

## Abstract

**Objective:**

Emerging evidence highlights the involvement of long non‐coding RNAs (lncRNAs) and ferroptosis in the pathogenesis of traumatic brain injury (TBI). However, the regulatory role of lncRNAs in TBI‐induced ferroptosis remains poorly understood. This study aims to investigate the role of a specific lncRNA, noncoding transcript of chemokine (C‐C motif) ligand 4 (*Ccl4*) overlapping (*Ntoco*), in the regulation of ferroptosis following TBI and explore its potential as a therapeutic target.

**Methods:**

The expression levels of *Ntoco* following controlled cortical injury (CCI) in mice were measured using real‐time PCR. Behavioral tests post‐injury were assessed using the rotarod test and Morris water maze, and lesion volume was evaluated using micro‐MRI. *Ntoco* binding proteins were identified using RNA pull‐down and RNA immunoprecipitation. RNA sequencing was employed to identify *Ntoco*‐related pathways. Western blotting and co‐immunoprecipitation were used to measure protein levels and ubiquitination processes.

**Results:**

*Ntoco* upregulation was observed in CCI mice. *Ntoco* knockdown inhibited neuron ferroptosis, reduced lesion volume, and improved spatial memory following TBI. *Ntoco* overexpression promoted ferroptosis in neurons. Mechanistically, *Ntoco* facilitated K48‐linked ubiquitination and degradation of proteins by binding to Hnrnpab, suppressing the NF‐κB/Lcn2 signaling pathway. This included reduced phosphorylation of IkBα, increased phosphorylation of IKKα/β, nuclear translocation of the NF‐κB p65 subunit, and elevated Lcn2 expression.

**Conclusion:**

Our findings suggest that *Ntoco* plays a crucial role in TBI‐induced ferroptosis by modulating the NF‐κB/Lcn2 signaling pathway. Targeting *Ntoco* may provide a promising therapeutic strategy to mitigate ferroptosis and improve outcomes following TBI.

## Graphical abstract

The study identifies *Ntoco* as a critical regulator of ferroptosis in traumatic brain injury (TBI). *Ntoco* knockdown alleviates neuronal damage, while its overexpression exacerbates ferroptosis. This research highlights targeting *Ntoco* as a potential therapeutic strategy for reducing ferroptosis‐induced damage in TBI.

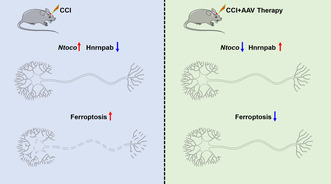

## 1 Introduction

Traumatic brain injury (TBI) is considered a global health priority, with approximately 55 million individuals affected worldwide [1] and an estimated annual economic burden of $400 billion [2]. During the past 30 years, the age‐standardized prevalence of TBI has increased by 8.4% [1], making it the leading cause of mortality and disability among young adults. The pathological mechanisms underlying TBI is complex, involving multiple processes, such as hemorrhage, local hypoxia/reoxygenation, secretion of pro‐inflammatory factors, excitotoxicity, oxidative stress, and neuronal death [3].

Ferroptosis, a recently discovered type of programmed cell death, is characterized by the accumulation of peroxidized lipids, an imbalance in reactive oxygen species, and iron overload [4, 5]. Biomarkers of ferroptotic cell death, such as acyl‐CoA synthetase long‐chain family member 4 (Acsl4), solute carrier family 7 member 11 (Slc7a11, also known as xCT), and spermidine/spermine N1‐acetyl transferase 1 (Sat1) are differentially altered in a series of diseases, including TBI. Therefore, anti‐ferroptosis might be a potential therapeutic strategy for treating TBI [6, 7, 8, 9, 10]. Deferoxamine, an iron chelator, has been reported to improve neurological function by suppressing ferroptosis and neuroinflammation and activating the nuclear factor kappa B (NF‐κB) pathway after TBI [11]. The activation of NF‐κB might result in upregulation of lipocalin 2 (LCN2), which depletes ferrous iron and induces resistance to ferroptosis [12]. In our previous work, we found that miR‐212‐5p might protect against ferroptotic neuronal death and improve spatial memory in TBI mice by targeting *Ptgs2* mRNA (encoding prostaglandin‐endoperoxide synthase‐2) [9].

Apart from microRNAs (miRNAs), long non‐coding RNAs (lncRNAs) have also been recognized as important neuromodulators in TBI [13, 14]. LncRNAs are defined as RNAs longer than 200 nucleotides (nt) that do not encode proteins [15, 16]. Due to the lack of protein‐coding potential, lncRNA transcripts were traditionally annotated as “transcriptional noise” [15]. However, recent evidence has shown that lncRNAs play critical roles in transcriptional and post‐transcriptional gene regulation by sequestering proteins [16, 17, 18, 19], and are involved in many diseases, including TBI [13, 20, 21, 22]. For example, lncRNA nuclear‐enriched abundant transcript 1 (*Neat1*) inhibits apoptosis and inflammation by capturing p53‐induced death domain‐containing protein 1, causing functional improvement in mice after TBI [13]. LncRNA metastasis‐associated lung adenocarcinoma transcript 1 (*MALAT1*) in exosomes modulates multiple targets following TBI, particularly those involved in inflammation‐related pathways [14]. Nevertheless, our knowledge of ferroptosis‐related lncRNAs in TBI remains limited.

In this study, we analyzed the lncRNA expression profile based on data from the Gene Expression Omnibus (GEO) database [23] and found that a noncoding transcript of chemokine (C‐C motif) ligand 4 (*Ccl4*) overlapping (abbreviated as *Ntoco* thereafter) was significantly upregulated in mice after TBI. Downregulation of *Ntoco* protected neurons against ferroptosis and improved TBI outcomes by activating the NF‐κB/Lcn2 signaling axis, suggesting that *Ntoco* might be a potential therapeutic target for TBI.

## 2 Material and Methods

### 2.1 Animals

The mouse strains used in this study were C57BL/6, aged 8–12 weeks, weighing 18–22 g, with an equal distribution of males and females. The mice were purchased from Dashuo (Chengdu, China) and housed in the animal facility of the West China School of Basic Medical Sciences and Forensic Medicine, Sichuan University. They were maintained on a 12‐h light/dark cycle and provided standard food and water ad libitum. The bedding was changed twice a week. Upon arrival, all animals underwent a 1‐week acclimation period before the start of the experimental procedures. All animal experiments in this study were approved by the Animal Ethics Committee of the West China Second University Hospital, Sichuan University, with approval number 2020 (015).

### 2.2 Animal Models

The experimental procedure involved subjecting mice to a controlled cortical injury (CCI) with craniotomy, following our previously published method [9]. Mice were anesthetized with isoflurane and secured in a stereotaxic frame. A craniotomy was performed over the left parietotemporal cortex, and a cortical impact was delivered with a strike velocity of 5.0 m/s, a strike depth of 2.0 mm, and a dwell time of 100 ms. The incision was closed, and mice with dural lacerations were excluded from the study. After a designated survival period, mice were anesthetized and perfused with isotonic saline. The ipsilateral cortex was then dissected and preserved at −80°C.

### 2.3 Intracerebroventricular Injection and Behavioral Tests

The AAV particles containing *Ntoco* shRNA or negative control were ordered from Genechem (Shanghai, China). Anesthetized mice were positioned within a stereotaxic frame, and 5 μL of 1 × 10^13^ UI AAV particles were delivered into each lateral ventricle using a 5 μL‐gauge Hamilton syringe at a rate of 1 μL/min over a duration of 10 min. To ensure optimal concentration and efficacy, the injections were administered 28 days prior to the CCI procedure.

The rotarod apparatus (Yuyan instruments, Shanghai, China) was cleaned and disinfected before each test. Mice were allowed to acclimate to the testing room for 15 min prior to testing. Each rotarod test lasted 300 s, starting at 0 rpm and gradually increasing to 30 rpm over 200 s. The recording time stopped when the mouse fell off the rotarod. Tests were conducted daily between 10 AM and 12 AM, with each mouse undergoing one test per day for three consecutive days. The results were collected and analyzed.

The Morris water maze (MWM) test consisted of two parts: the place navigation test and the spatial probe trial, following our previous method [9]. The water maze equipment was manufactured by Techman (Chengdu, China). Mice were placed in a circular tank filled with water (diameter 60 cm, depth 30 cm, and temperature 23°C ± 1°C) mixed with an opaque dye. An escape platform was submerged 1.5 cm below the water surface. The movement trajectory and escape latency of the mice were recorded. After 7 days, the escape platform was removed, and mice were allowed to explore the tank for 4 min. Their movement trajectory and dwell time in each area were recorded. The escape latency and the number of platform crossings were analyzed statistically.

### 2.4 Cell Culture and Transfection

HT22 cell lines were cultured in high‐glucose Dulbecco's modified Eagle's medium (DMEM) (Hyclone, Logan, UT, USA) supplemented with 10% fetal bovine serum (FBS, Hyclone) and 1% penicillin/streptomycin (P/S, Hyclone) at 37°C with 5% CO_2_. For primary neuronal cultures, cortical tissues from newborn mice were dissected and digested with papain, following a previously described method [24]. The dissociated cells were resuspended in Neurobasal‐A medium (Thermo Fisher Scientific, Waltham, MA, USA) supplemented with 2% B27 (Thermo Fisher Scientific), 0.5 mM glutamine (Saiguo Biotech, Guangzhou, China), and 0.5% P/S (Hyclone). The cells were plated on poly‐l‐lysine‐coated dishes and maintained at 37°C with 5% CO_2_. The culture medium was changed to a feeding medium every 3 days. For cell transfection, when the cell confluency reached 50%–75%, 5 μL of 1 × 10^9^ UI Lentivirus particles (Genechem), 2.5 μg plasmid DNA, or 100 mM siRNA (transfected with Lipofectamine 3000) were added to each 2 mL well. Transfection efficiency was observed 2–3 days post‐transfection.

### 2.5 Quantitative Real‐Time Reverse Transcription PCR (qRT‐PCR) Assay

RNA of cells or tissues was extracted using a fast extraction kit (Abclonal, Wuhan, China). The quality and concentration of the extracted RNA were assessed using a NanoPhotometerN60 (IMPLEN, Westlake Village, CA, USA), with the A260/A280 value within the range of 1.9–2.1. One microgram of RNA was reverse‐transcribed into cDNA using the RevertAid First Strand cDNA Synthesis Kit (Thermo Fisher Scientific) with random primers. The resulting cDNA was subjected to qPCR with the primers summarized in Table S1. Each sample underwent triplicate runs in a 10 μL reaction containing QuantiNova SYBR Green PCR Kit (Qiagen, Hilden, Germany). The reactions were conducted on a Cobas Z 480 Light Cycler (Roche Diagnostics, Mannheim, Germany). The relative RNA expression levels in the treated groups compared to the control group were calculated using the Δ*C*_t_ method ($2^{-∆∆C_{t}}$). All data were normalized to the level of *Actb* (encoding β‐actin).

### 2.6 Rapid Amplification of cDNA Ends (RACE)

Fragments of *Ntoco* were amplified using a 5′‐RACE kit (Roche Diagnostics) and a 3′‐RACE kit (Thermo Fisher Scientific). The 5′‐RACE technique was employed according to the manufacturer's instructions to amplify the 5′ end of the cDNA. For the 3′‐RACE analysis, an adapter was ligated to the 3′‐hydroxyl group of the RNA, followed by specific amplification using gene‐ and adapter‐specific primers. The PCR products obtained from RT‐PCR, 5′‐RACE, and 3′‐RACE were extracted, purified, and subjected to electrophoresis analysis, followed by DNA sequencing for verification.

### 2.7 Flow Cytometry

Mouse cerebral cortex and hippocampal tissues were digested using papain and filtered to obtain a single‐cell suspension. The cells were fixed with 4% paraformaldehyde for 15 min. After centrifugation, the cells were resuspended in methanol and incubated on ice. Primary antibodies were added and incubated for 1 h at room temperature. Following centrifugation, a fluorescent secondary antibody was added and incubated in the dark for 30 min. The cells were then resuspended in phosphate‐buffered saline (PBS), filtered into a flow tube, and sorted by flow cytometry based on size and fluorescence categories.

### 2.8 Plasmid Constructs

In the RNA pull‐down experiment involving the *Ntoco* antisense chain and its fragments, the pBluescript II SK+ plasmid was used as the cloning vector for the in vitro transcription template. Specific restriction endonucleases, *Kpn* I and *Apa* I, were employed for vector digestion. All ligation reactions were carried out using T4 ligase (New England Biolabs, Ipswich, MA, USA) according to the manufacturer's instructions. Plasmid sequencing verification was performed to ensure the successful ligation of vectors.

### 2.9 Cytotoxicity Assay

A CytoTox 96 non‐radioactive cytotoxicity assay kit (Promega, Madison, WI, USA) was used to measure lactate dehydrogenase (LDH) release as an indicator of cell death. Cells were seeded at a density of 5000 cells per well in a 96‐well plate and treated with 3 μM RAS‐selective lethal 3 (RSL3). After 24 h, the supernatant from the background, lysis, and experimental wells was transferred to a new plate, followed by the addition of a working solution. The plate was incubated at 37°C for 30 min, and the optical density (OD) value at 492 nm was measured. The LDH release rate was calculated using the formula: LDH release rate (%) = (mean OD of experimental wells—mean OD of background wells)/(mean OD of lysis wells—mean OD of background wells) × 100%.

### 2.10 JC‐1 Mitochondrial Membrane Potential and Intracellular Iron Ion Measurement Detection

The JC‐1 fluorescent probe and FerroOrange fluorescent probe (Dojindo, Kumamoto, Japan) were used to detect mitochondrial membrane potential and Fe^2+^ levels, respectively. Cells were seeded at a density of 5000 per well in a 96‐well plate. After 24 h, the culture medium was replaced with RSL3‐containing medium, followed by incubation for an additional 24 h. According to the manufacturer's instructions, a 4 μM JC‐1 or FerroOrange working solution was added and incubated for 1 h or 30 min, respectively. The cells were then washed and observed immediately using a fluorescence microscope.

### 2.11 Perls' Stain and Mitochondrial Morphology Assay

A Perls' staining kit (Solarbio, Beijing, China) was used to stain brain slices from mice after CCI. The brain tissue sections were incubated with Perls' staining working solution at 37°C for 30 min. After rinsing and re‐staining, the sections were dried, sealed, and examined under a microscope for photography.

For transmission electron microscopy (TEM) (Jeol, Akishima, Tokyo, Japan), brain tissues from sham and CCI mice and cells treated with RSL3 were fixed with 4% paraformaldehyde and stored at 4°C. Both tissue and cell specimens were sent to the Lilai Biomedicine Experimental Center (Chengdu, China) for sectioning and TEM analysis.

### 2.12 RNA Pull‐Down

*Ntoco* was transcribed using a T7 high‐yield RNA transcription kit (Vazyme Biotech, Nanjing, China). Biotinylation was performed at the 3′ end of *Ntoco* using a Pierce RNA 3′ end desthiobiotinylation kit (Thermo Fisher Scientific). HT22 cell lysate with a protein concentration > 2 mg/μL was prepared. Streptomycin affinity magnetic beads from the Pierce magnetic RNA‐protein pull‐down kit (Thermo Fisher Scientific) were used. *Ntoco* was incubated with the magnetic beads overnight at 4°C, and the bound proteins were subsequently eluted. The eluted products were analyzed by silver staining and mass spectrometry at Genecreate Biological Engineering (Wuhan, China).

### 2.13 RNA Immunoprecipitation (RIP)

RIP was performed using a Magna RIP RNA‐binding protein immunoprecipitation kit (Millipore, Bedford, MA, USA). Following the manufacturer's instructions, HT22 cells were lysed, and 5 μg/mL of antibodies specific to Hnrnpab or IgG (Abcam) were added. Protein A/G magnetic beads were used for immunoprecipitation. After washing, the bound RNA was eluted, purified, and reverse transcribed. qPCR analysis was performed using primers specific to *Ntoco*, as well as control primers for *GAPDH*, *Malat1*, and *Neat1*.

### 2.14 Co‐Immunoprecipitation (Co‐IP)

A Pierce classic IP Kit (Thermo Fisher Scientific) was used to detect protein binding according to the methods described previously [25]. HT22 cells with and without *Ntoco* overexpression were lysed on ice using lysis buffer. The cell lysate was added to reaction tubes containing anti‐Hnrnpab antibodies or IgG (2 μg/mL, Abcam). After incubation for 1 h at 4°C, protein A/G magnetic beads were added and incubated again. The magnetic beads were washed and separated, and the supernatant was discarded. Finally, a protein loading buffer was added, and the supernatant was separated by sodium dodecyl sulfate‐polyacrylamide gel electrophoresis (SDS‐PAGE) and detected using western blotting with anti‐ubiquitin or anti‐k48‐ubiquitin antibodies (Abcam).

### 2.15 Western Blotting

Cells were lysed on ice using Radioimmunoprecipitation assay (RIPA) lysis buffer (Labselect) to extract proteins. Tissues were sheared, ground, and lysed on ice using RIPA lysis buffer. After centrifugation at 4°C for 20 min at 1000 *g*, the supernatant was collected. Protein concentration was determined using a bicinchoninic acid (BCA) kit (Yeasen Biotechnology, Shanghai, China). Protein samples were boiled for 5 min with 1 × loading buffer, separated by SDS‐PAGE, and transferred onto polyvinylidene fluoride membranes (Bio‐Rad, Hercules, CA, USA). The membranes were blocked with 5% skim milk, incubated with primary antibodies overnight at 4°C, and then with secondary antibodies at room temperature for 1 h. Antibody information is provided in Table S2. The membranes were washed using 1 × Tris‐buffered saline with 0.1% Tween 20 (TBST) buffer and visualized using enhanced chemiluminescence (ECL), followed by photographic documentation.

### 2.16 Micro‐Magnetic Resonance Imaging (MRI) Assay

T1‐weighted MRI of mice was performed using a Nova 7.0T (Time Medical Systems, Burlingame, CA, USA) on days 0, 3, and 7 post‐CCI. The scans encompassed 12 coronal sections of the entire brain. Mice were anesthetized with 2% isoflurane and positioned on the MRI scanning bed. Following image acquisition, the midline‐positioned images were organized and analyzed statistically.

### 2.17 Hematoxylin and Eosin (HE) Staining

Tissue samples were fixed in 4% paraformaldehyde, dehydrated through an ethanol gradient, embedded in paraffin, and sectioned into 4 μm thick slices. The sections were deparaffinized, rehydrated, and stained with hematoxylin for 5 min to visualize nuclei, followed by eosin staining for 2 min to highlight the cytoplasm. After dehydration and mounting, the slides were observed and imaged under a light microscope.

### 2.18 Bioinformatic Analyses

The catRAPID online tool (http://s.tartaglialab.com/page/largeRNAs_group) and its Global Score algorithm were used to assess the binding tendency between *Ntoco* and Hnrnpab protein [26]. The 3dRPC web server (http://biophy.hust.edu.cn/new/3dRPC) and the mol* 3D viewer of the protein data bank (https://www.rcsb.org/3d‐view) were used for the 3D structure prediction of the *Ntoco*‐Hnrnpab complex [27, 28]. DESeq2 software was used for gene normalization and differential analysis of the RNA‐sequencing data. Differentially expressed protein‐coding genes were selected based on fold change (FC) and significance tests. The OECloud tools software was used for gene expression analysis, and the volcano plot was generated using a significance threshold of *p* < 0.05 and a threshold of |FC| > 2. Kyoto Encyclopedia of Genes and Genomes (KEGG), a prominent pathway database [29], was used for pathway analysis of differentially expressed protein‐coding genes. Enrichment analysis was performed using the hypergeometric distribution test, and the significance of the enriched differentially expressed genes in each pathway was calculated. The resulting KEGG‐enriched pathways were ranked based on the number of differentially expressed genes (> 2) and *p*‐value and visualized using a bubble chart.

### 2.19 Statistical Analysis

All data were confirmed to follow a normal distribution. Continuous data are presented as the mean ± standard error of the mean (SEM) and were analyzed using Student's *t*‐test, one‐way analysis of variance (ANOVA), or repeated measures ANOVA, as appropriate. Statistical analyses were performed using SPSS Statistics 13.0 software (IBM Corp., Armonk, NY, USA), with a threshold of *p* < 0.05 indicating statistical significance. Graphs were generated using GraphPad Prism 9 (GraphPad Inc., La Jolla, CA, USA).

## 3 Results

### 3.1 LncRNA *Ntoco* Expression Is Significantly Increased in CCI Mice

We analyzed the differential expression profile of lncRNAs following CCI in mice, based on data from the GEO (GSE79441) [23]. The following criteria were used for lncRNA identification: (1) A Reads Per Kilobase per Million mapped reads (RPKM) value > 0; (2) Full‐length lncRNA < 3000 nt; (3) FC of lncRNA overexpression > 6.0 in the CCI group compared to the sham group; (4) Adjusted *p* value < 0.001 in the CCI group versus the sham group. Finally, only *Ntoco*, which was highly expressed in the cortex of CCI mice, was selected for further analysis (Figure S1).

**FIGURE 1:**
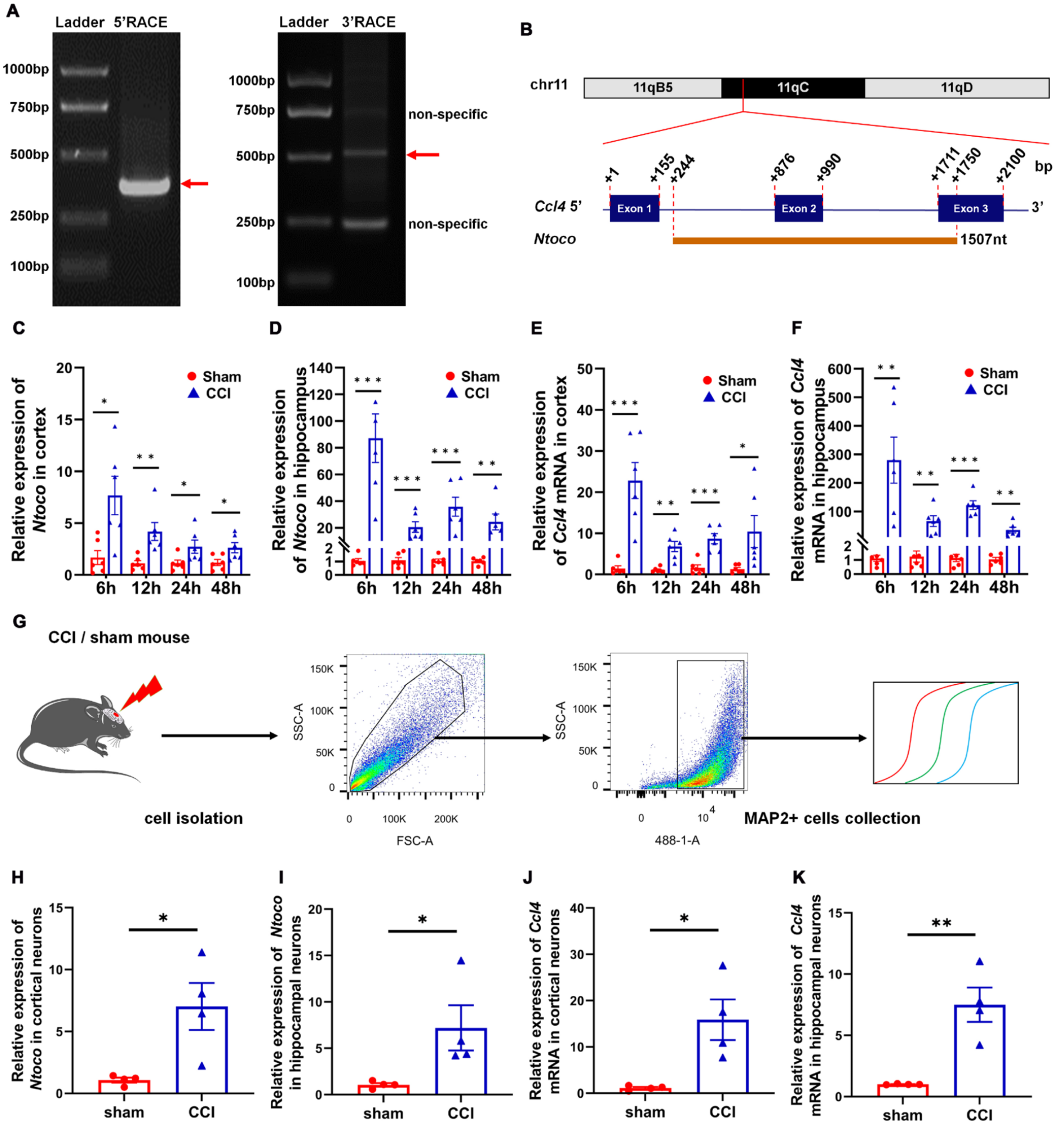
LncRNA *Ntoco* expression is significantly increased in CCI mice. (A) Agarose gel imaging for 5′‐RACE (left) and 3′‐RACE (right) products. (B) Schematic diagram showing the full length of *Ntoco* and its relationship with *Ccl4*. (C–F) *Ntoco* and *Ccl4* mRNA levels in the cortex and hippocampus of sham and CCI mice at 6–48 h post‐injury (*n* = 6). (G) Fluorescence‐activated cell sorting (FACS) was used to isolate MAP2 (+) neurons from the cortex and hippocampus of sham and CCI mice. (H–K) *Ntoco* and *Ccl4* mRNA levels in FACS‐isolated cortical and hippocampal neurons of sham and CCI mice at 24 h post‐injury (*n* = 4). *p < 0.05, **p < 0.01, ***p < 0.001 versus the sham group using Student's *t*‐test.

A RACE assay was performed to identify the full length of *Ntoco*. The 5′‐RACE generated a single band, while the 3′‐RACE produced three bands, two of which were non‐specific (Figure 1A). As shown in Figure 1B, *Ntoco* mapped to exon 2, intron 2, part of intron 1, and exon 3 of *Ccl4*, with a full length of 1507 nt (chr11:83476329–83477835 (+)) (Figure S2).

Compared to the sham injury group, the expression levels of *Ntoco* and *Ccl4* mRNA were increased at 6–48 h following CCI in the cortex and hippocampus of mice (Figure 1C–F). To determine whether the differential expression occurs in specific neurons, flow cytometry was used to sort cortical and hippocampal neurons from sham and CCI mice (Figure 1G). The results demonstrated significantly higher expression of *Ntoco* and *Ccl4* mRNA in cortical and hippocampal neurons after CCI compared to the sham injury group (Figure 1H–K). Overall, these findings indicate that *Ntoco* is significantly upregulated in mice after CCI.

### 3.2 *Ntoco* Promotes RSL3‐Induced Ferroptosis

**FIGURE 2:**
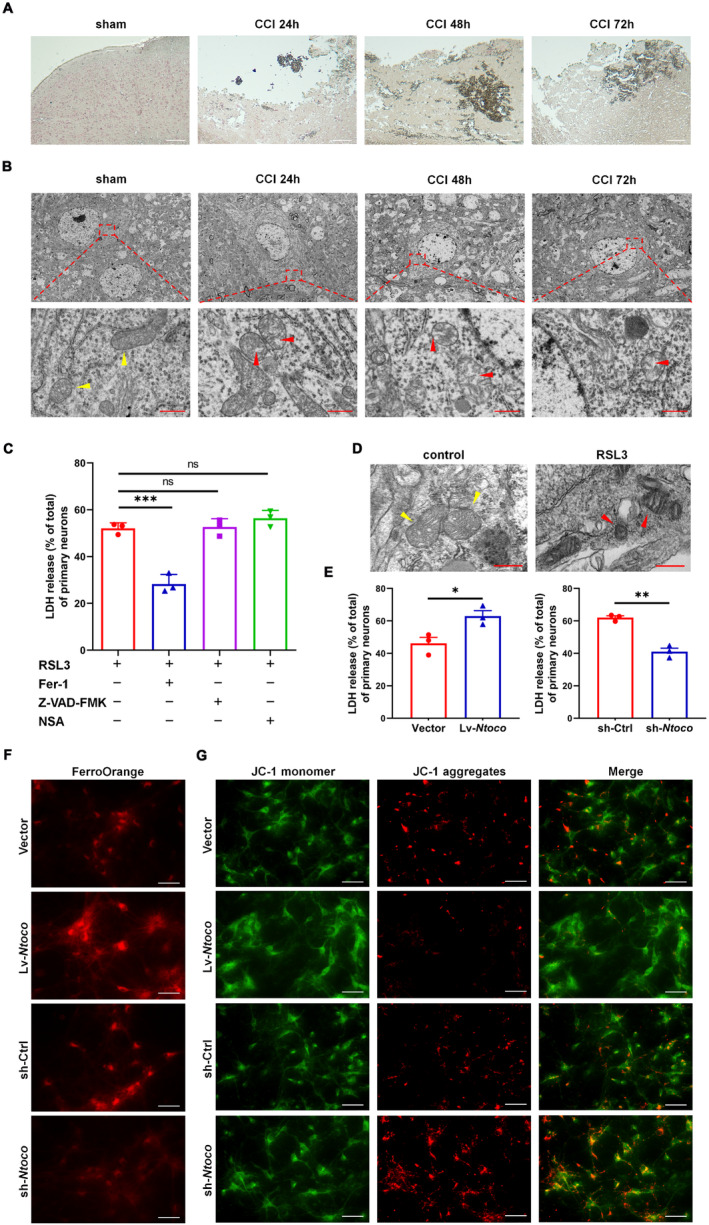
*Ntoco* promotes RSL3‐induced ferroptosis. (A) Perls' blue staining of brain sections from sham and CCI mice at 24–72 h post‐injury. Scale bar: 100 μm. (B) Transmission electron microscopy (TEM) images of brain sections from sham and CCI mice at 24–72 h after injury. The yellow arrow indicates normal mitochondria. The red arrow indicates CCI‐induced mitochondrial damage. Scale bar: 500 nm. (C) Lactate dehydrogenase (LDH) cytotoxicity assay showing the LDH release rate in primary neurons treated with RSL3 (3 μM), the ferroptosis inhibitor ferrostatin‐1 (Fer‐1, 0.4 μM), the apoptosis inhibitor zVAD‐fmk (10 μM), or the necroptosis inhibitor necrosulfonamide (NSA, 0.5 μM). (D) TEM images of primary neurons treated with and without RSL3. The yellow arrow indicates normal mitochondria. The red arrow indicates RSL3‐induced mitochondrial damage. Scale bar: 500 nm. (E–G) Primary neurons with *Ntoco* overexpression (Lv‐*Ntoco*) or knockdown (sh‐*Ntoco*) were treated with RSL3. After 24 h, the LDH release rate, FerroOrange, and JC‐1 staining were used to measure RLS3‐induced cytotoxicity (E), ferrous ion levels (F) (scale bar: 20 μm), and mitochondrial membrane potential (G) (scale bar: 10 μm), respectively. *p < 0.05 versus the overexpression control (vector), **p < 0.01 versus the downregulation control (sh‐Ctrl) using Student's *t*‐test.

Our previous work showed that the concentration of malondialdehyde (MDA), ferrous ions, and the expression of ferroptosis‐related molecules, such as NADPH oxidase 2 (Nox2), xCT, and Sat1 were increased in mice following CCI [9]. In this study, we used Perls' blue and TEM to visualize the morphological characteristics of ferroptosis in CCI mice. As shown in Figure 2A,B, an accumulation of iron‐positive cells, condensed mitochondrial membrane density, and reduced mitochondrial cristae were observed at 24–72 h post‐injury. These findings suggest that CCI induces ferroptotic neuronal death.

In vitro gain‐ and loss‐of‐function experiments were performed to determine whether *Ntoco* influences ferroptosis. We first used RSL3 to induce cell death and found that RSL3‐induced cell death was reversed by treatment with the ferroptosis inhibitor ferrostatin‐1 but not by the apoptosis inhibitor zVAD‐fmk or necroptosis inhibitor necrosulfonamide (Figure 2C and Figure S3A,B). TEM showed that cells exposed to RSL3 exhibited an abnormal mitochondrial ultrastructure, such as mitochondrial shrinkage, increased mitochondrial membrane density, and vanishing of mitochondrial cristae (Figure 2D and Figure S3C). The expression of ferroptosis‐related genes, including *Acsl4*, *Sat1*, and *Slc7a11*, was detected by qRT‐PCR, showing significant upregulation following RSL3 treatment (Figure S3D). These findings confirm that RSL3 successfully induced ferroptosis.

We then investigated the role of *Ntoco* in ferroptosis by constructing lentivirus‐delivered overexpression and antisense oligonucleotide of *Ntoco*. The efficiency of overexpression and knockdown of *Ntoco* is shown in Figure S4A–D. Overexpression of *Ntoco* promoted RSL3‐induced cytotoxicity and mitochondrial depolarization and enhanced intracellular Fe^2+^ levels. In contrast, the knockdown of *Ntoco* alleviated RSL3‐induced cytotoxicity and mitochondrial depolarization and decreased intracellular Fe^2+^ levels (Figure 2E–G and Figure S5A–C). The effects of *Ntoco* on the genetic hallmarks of ferroptosis (i.e., *Acsl4*, *Slc7a11*, and *Sat1*) were evaluated using qRT‐PCR. The results showed that the upregulation of *Ntoco* significantly increased *Acsl4*, *Slc7a11*, and *Sat1* expression, whereas *Ntoco* silencing had the opposite effect (Figure S6A,B). Altogether, these findings indicate that *Ntoco* promotes RSL3‐induced ferroptosis.

### 3.3 *Ntoco* Binds to Hnrnpab and Induces K48‐Linked Ubiquitination and Protein Degradation

**FIGURE 3:**
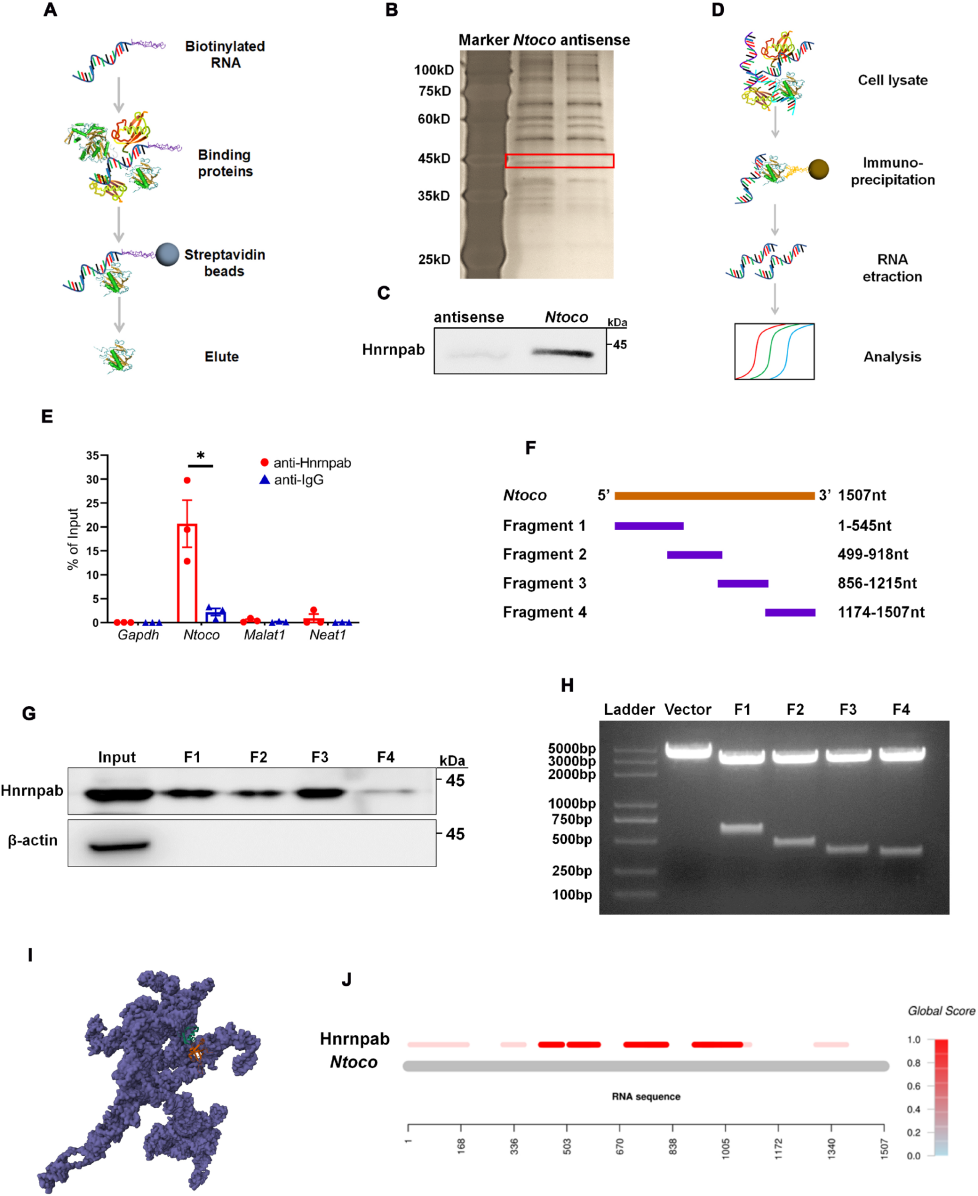
*Ntoco* binds to Hnrnpab. (A) Flowchart of *Ntoco*‐binding protein analysis. (B) Sodium dodecyl sulfate polyacrylamide gel electrophoresis and silver staining analysis of the interacting protein pulled down by biotinylated *Ntoco* and antisense transcripts. (C) Western blotting confirms the binding of *Ntoco* and Hnrnpab. (D) Flowchart of RNA binding protein immunoprecipitation (RIP)‐qRT‐PCR analysis. (E) *Ntoco* enrichment immunoprecitated by rabbit anti‐Hnrnpab antibody was analyzed using qRT‐PCR. *p < 0.05 versus the IgG group using Student's *t*‐test. (F) Diagram of full‐length *Ntoco* and its fragments used for RNA‐protein pull‐down analysis. (G) Agarose gel electrophoresis image of reconstructed plasmids containing full‐length and fragments 1–4 (F1–F4) of *Ntoco*. (H) Western blotting revealing the binding of *Ntoco* F1–4 and Hnrnpab. (I) The three‐dimensional structure of the *Ntoco*–Hnrnpab complex was predicted using 3dPRC software. The purple indicates *Ntoco*, and the orange‐green fold indicates Hnrnpab. (J) The interaction propensity between *Ntoco* and Hnrnpab was predicted using catRAPID software, with the depth of the red color indicating the interaction strength of the *Ntoco*‐Hnrnpab pair.

LncRNAs are known to achieve regulatory specificity by interacting with RNA‐binding proteins (RBPs) [30]. In this study, we used an RNA pull‐down assay to identify *Ntoco‐*binding proteins. Specifically, sense and antisense transcripts of *Ntoco* were biotinylated and incubated with HT22 cell lysates. After isolation from streptavidin magnetic beads, the eluted RNA‐binding protein complexes were analyzed by SDS‐PAGE (Figure 3A). A protein around 45 kDa that specifically binds to *Ntoco* was observed and identified as heterogeneous nuclear ribonucleoprotein A/B (Hnrnpab) using mass spectrometry (Figure 3B and Figure S7A,B), which was further verified by western blotting (Figure 3C). Moreover, a *Ntoco* fragment immunoprecipitated by anti‐Hnrnpab antibodies was detected using the RIP assay (Figure 3D,E). To locate the specific binding region between *Ntoco* and Hnrnpab, a series of *Ntoco* truncations were established for an RNA pull‐down assay (Figure 3F,G). We found that nucleotides 1 to 1215 of *Ntoco* bound to Hnrnpab (Figure 3H), consistent with predictions from an online software (Figure 3I,J). Together, the RNA pull‐down and RIP experiments demonstrate the interaction of *Ntoco* with Hnrnpab.

**FIGURE 4:**
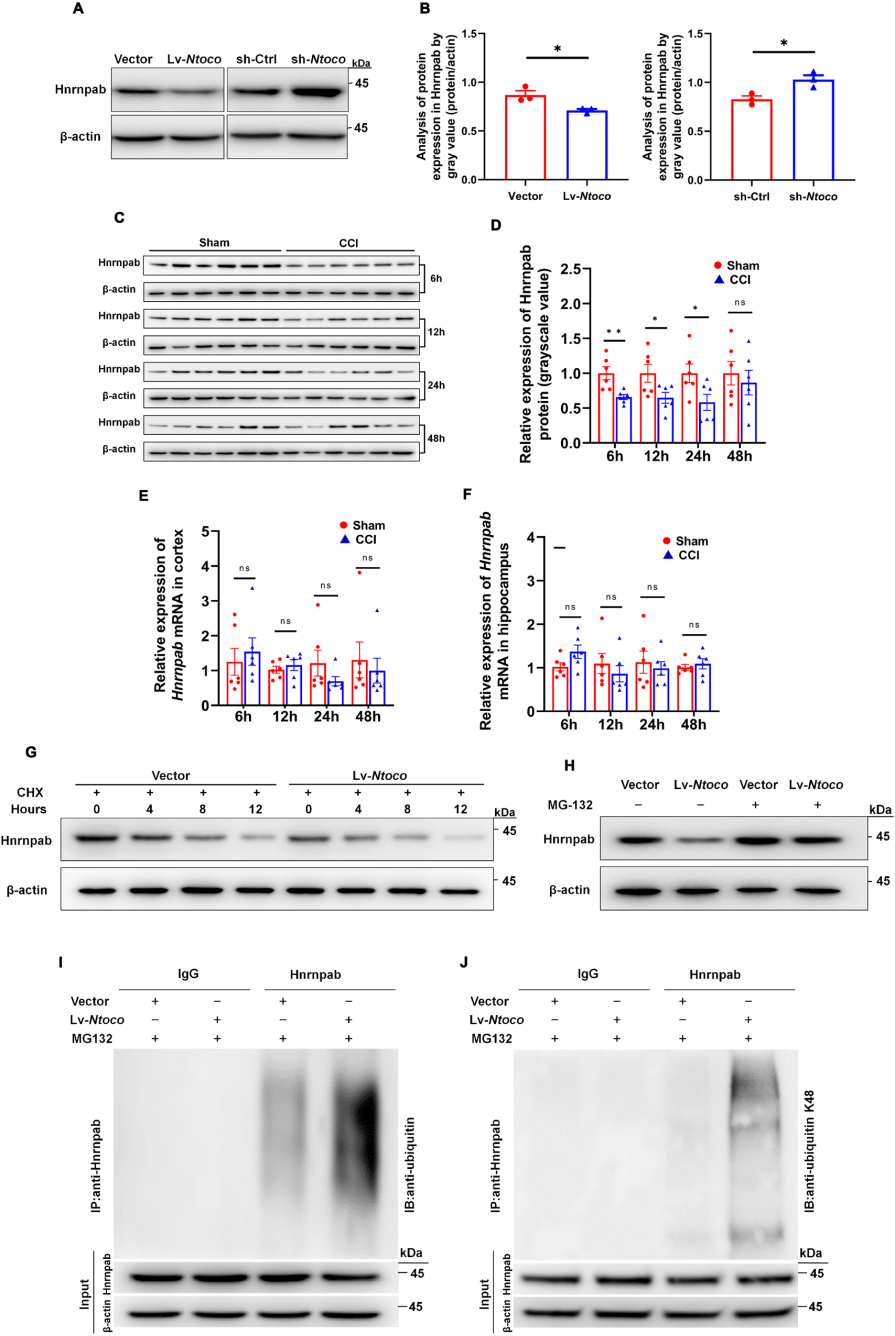
*Ntoco* induces K48‐linked ubiquitination and Hnrnpab degradation. (A, B) Hnrnpab levels in *Ntoco* overexpression (Lv‐*Ntoco*) and knockdown (sh‐*Ntoco*) HT22 cells. Vector, overexpression control; sh‐Ctrl, downregulation control. At 6–48 h after CCI, the relative expression of Hnrnpab protein and mRNA was detected using western blotting (C, D) and qRT‐PCR (E, F), respectively. (G) The effect of *Ntoco* overexpression on the half‐life of Hnrnpab was examined in HT22 cells treated with cycloheximide (CHX, 50 μg/mL) at the indicated time points. (H) Hnrnpab levels in *Ntoco*‐overexpressing HT22 cells with and without MG‐132 treatment. (I, J) The effect of *Ntoco* overexpression on the levels of ubiquitination and K48‐linked ubiquitination of Hnrnpab was examined using immunoprecipitation in HT22 cells treated with MG‐132 (10 μM) for 4 h. ns, not significant, *p < 0.05, **p < 0.01 versus the sham group using Student's *t*‐test.

We next examined whether *Ntoco* influences the expression of its binding protein, Hnrnpab, at the transcriptional and translational levels. As shown in Figure 4A,B, *Ntoco* overexpression decreased Hnrnpab levels, while *Ntoco* knockdown produced the opposite effect. Interestingly, the *Hnrnpab* mRNA levels were not significantly different between the groups regardless of *Ntoco* overexpression or downregulation (Figure S8A,B). Similar results were also observed in vivo, i.e., the protein level, but not the mRNA expression, of Hnrnpab was reduced in mice following 6–24 h of CCI (Figure 4C–F). Both the in vitro and in vivo results suggest that *Ntoco* might contribute to post‐translational modification, resulting in reduced Hnrnpab protein levels.

Protein degradation is a crucial contributor to the reduction in protein levels [31]. Therefore, we treated cells with the protein synthesis inhibitor cycloheximide (CHX) to determine whether *Ntoco* impacts Hnrnpab degradation. As shown in Figure 4G, the half‐life of Hnrnpab was notably shorter in *Ntoco*‐overexpressing cells than in the control cells, suggesting that *Ntoco* might enhance the proteasomal degradation of Hnrnpab. Considering the commonality and importance of the ubiquitin‐proteasome system (UPS) in post‐translational regulation and protein degradation [32], we hypothesized that Hnrnpab degradation is mediated by the UPS. As expected, we found that the decreased Hnrnpab level upon overexpression of *Ntoco* was reversed using the proteasome inhibitor MG‐132 (Figure 4H), supporting the role of the UPS in controlling Hnrnpab degradation. In line with these results, immunoprecipitation of ubiquitin followed by western blotting revealed that ubiquitination signals of Hnrnpab were significantly increased in *Ntoco*‐overexpressing cells (Figure 4I). K48 chains are the most common ubiquitylation pattern leading to proteasomal degradation [33]; therefore, we employed a co‐IP assay to determine whether K48 ubiquitylation participates in Hnrnpab degradation. Using antibodies targeting ubiquitin‐K48, we found that *Ntoco* enhanced K48‐specific ubiquitylation of Hnrnpab (Figure 4J).

### 3.4 Downregulation of *Ntoco* Activates the Hnrnpab‐Mediated NF‐κB/Lcn2 Signal Axis

**FIGURE 5:**
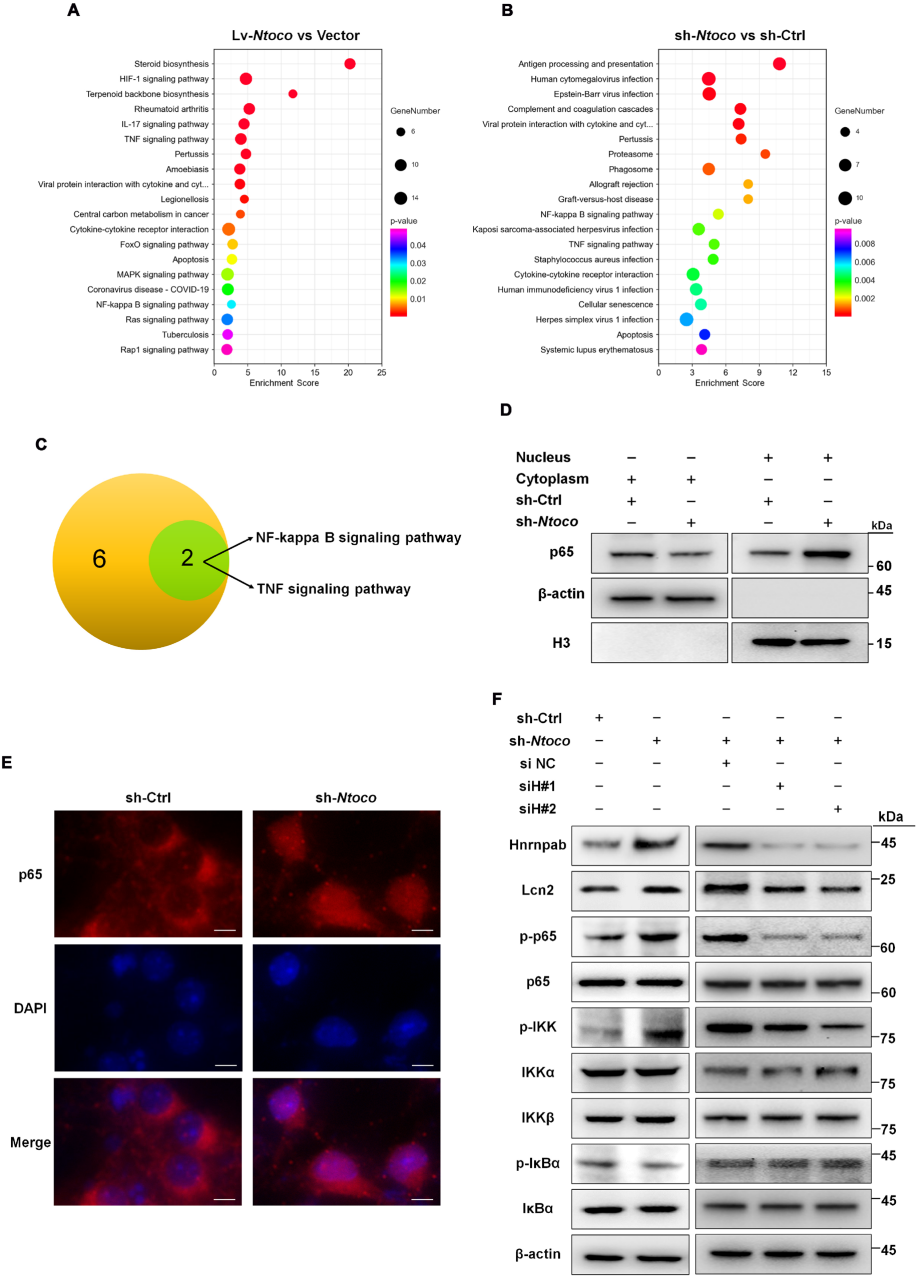
Downregulated *Ntoco* activates the Hnrnpab‐mediated NF‐κB/Lcn2 signaling axis. (A, B) Bubble chart visualizing KEGG pathway from RNA sequencing in *Ntoco* overexpressing (Lv‐*Ntoco*) and silenced (sh‐*Ntoco*) cells. (C) Intersection of signal *transduction* pathways related to *Ntoco* overexpression and downregulation. (D) Western blotting analysis of NF‐κB p65 subunit levels in the cytoplasmic and nuclear fractions of primary neurons with *Ntoco* knockdown. (E) Immunofluorescence analysis of p65 relocalization in primary neurons with *Ntoco* knockdown. Scale bar: 5 μm. (F) Left panel, protein levels of the NF‐κB/Lcn2 signaling axis in *Ntoco* knockdown cells. In the right panel, two validated small interfering RNAs against *Hnrnpab* (siH#1 and siH#2) were transfected into *Ntoco* knockdown cells. Western blotting was performed to analyze the NF‐κB/Lcn2 signaling axis protein levels.

To further explore the mechanism by which *Ntoco* downregulation inhibits ferroptosis, we carried out high‐throughput RNA sequencing and KEGG pathway enrichment analysis to identify *Ntoco*‐related signaling *transduction* pathways. In total, 151 differentially expressed genes were identified in *Ntoco* knockdown cells and were enriched for 30 signaling pathways, while 434 differentially expressed genes were identified in *Ntoco*‐overexpressing cells and were enriched for 39 signaling pathways (Figure 5A,B and Figure S9A,B). Among the pathways, eight from *Ntoco*‐overexpressing cells and two from *Ntoco* knockdown cells were related to signal *transduction, including two* intersection pathways: the NF‐κB signaling pathway and the tumor necrosis factor (TNF) signaling pathway (Figure 5C and Figure S9C), both of which were implicated in ferroptosis [12, 34].

The NF‐κB/Lcn2 axis is an important regulator of ferroptosis [12]. Therefore, we examined whether *Ntoco* regulates the NF‐κB/Lcn2 signaling axis. We found that *Ntoco* knockdown not only induced the nuclear translocation of the NF‐κB p65 subunit (Figure 5D,E and Figure S10A,B) but also significantly decreased the phosphorylation of NF‐κB inhibitor alpha (IkBα), increased the phosphorylation of inhibitor of NF‐κB subunit alpha and beta (IKKα/β) and p65, and increased Lcn2 expression (Figure 5F). To determine whether *Ntoco*‐mediated phosphorylation is Hnrnpab‐dependent, we used lentivirus containing Hnrnpab‐interfering (siHnrnpab) sequences to knock down *Hnrnpab*. Two out of three siHnrnpab sequences were found to decrease the mRNA and protein levels of Hnrnpab most efficiently (Figure S11A,B). As shown in Figure 5F, *Ntoco*‐mediated phosphorylation and Lcn2 levels were rescued by co‐transfection with siHnrnpab. Taken together, these findings suggest that downregulation of *Ntoco* activates the NF‐κB/Lcn2 signaling axis in an Hnrnpab‐dependent manner.

### 3.5 Knockdown of *Ntoco* Ameliorates Spatial Memory and CCI‐Induced Lesion Volume via Activating the NF‐κB/Lcn2 Signal Axis

**FIGURE 6:**
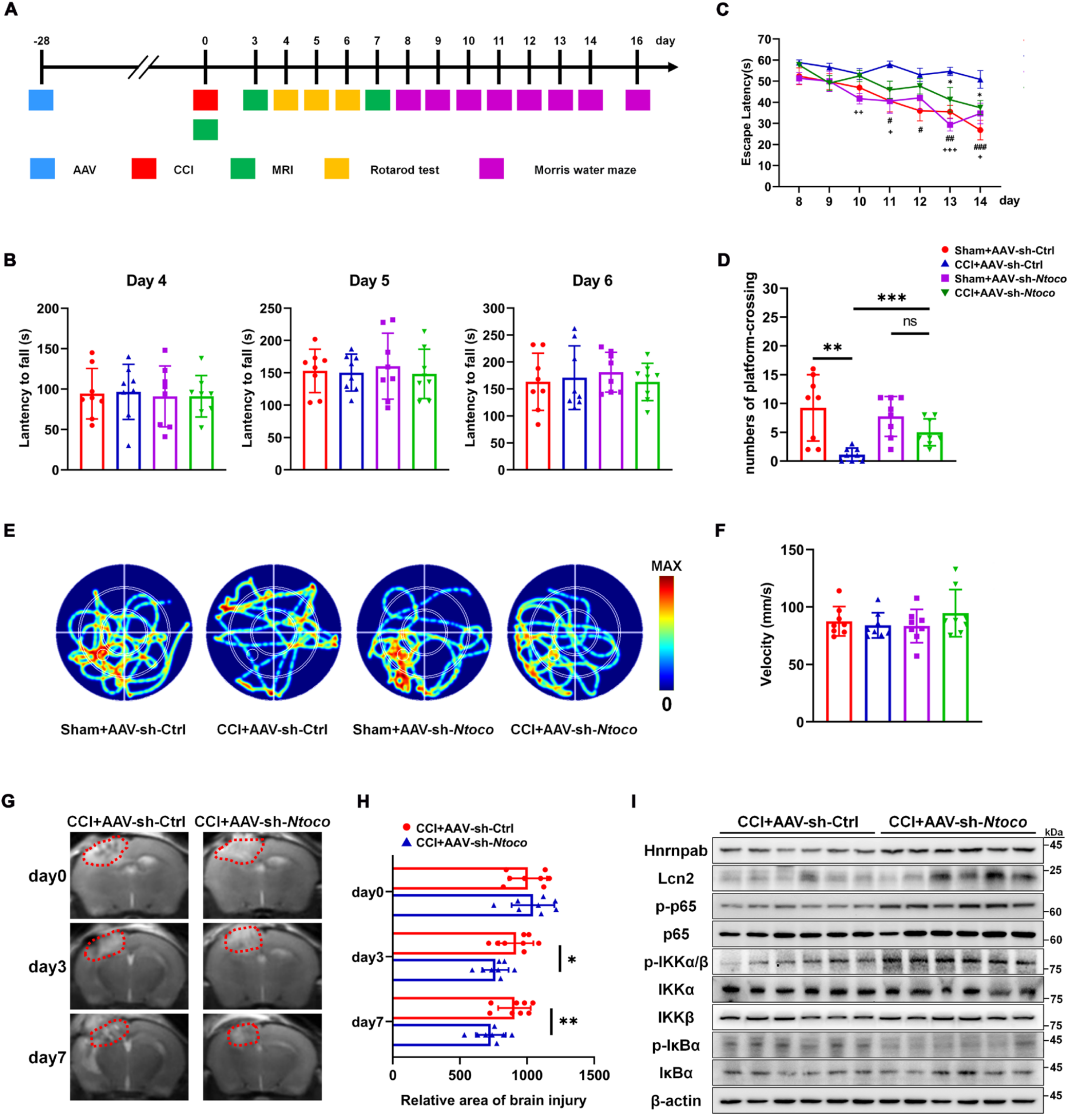
Knockdown of *Ntoco* ameliorates spatial memory and reduces CCI‐induced lesion volume by activating the NF‐κB/Lcn2 signaling axis. (A) Timeline diagram of neurobehavioral tests. AAV‐sh‐*Ntoco* and AAV‐sh‐Ctrl were microinjected into the bilateral ventricles 28 days prior to the injury. Magnetic resonance imaging (MRI) was utilized to evaluate the lesion volume on days 0, 3, and 7 post‐CCI. The rotarod test was used to assess motor function on days 4–6 post‐injury. The Morris water maze (MWM) was used to assess spatial memory acquisition (hidden platform testing) from days 8 to 14 and retention (probe trials) on the 16th day post‐CCI. (B) The latency to fall was not different among the groups, based on repeated measures ANOVA (*n* = 8). (C) The escape latency during hidden platform testing was plotted for each group (*n* = 8). *Indicates the CCI + AAV‐sh‐*Ntoco* group versus the CCI + AAV‐sh‐Ctrl group; ^#^Indicates the Sham+AAV‐sh‐Ctrl group versus the CCI + AAV‐sh‐Ctrl group; ^+^Indicates the Sham+AAV‐sh‐*Ntoco* group versus the CCI + AAV‐sh‐Ctrl group. *^,#,+^ *p* < 0.05, ^##,++^ *p* < 0.01, ^###,+++^ *p* < 0.001 versus the CCI + AAV‐sh‐Ctrl group by the repeated measures ANOVA. (D–F) The number of platform crossings, heatmap of swimming tracks, and swimming speed during the probe trial test were recorded (*n* = 8). Data were analyzed by the repeated measures ANOVA. ns, not significant; **p < 0.001, ***p < 0.001. (G, H) Representative MRI images of brain lesion volume (outlined in red) in CCI mice pre‐microinjected with AAV‐sh‐Ctrl or AAV‐sh‐*Ntoco* (*n* = 8). *p < 0.05, **p < 0.01 versus the CCI + AAV‐sh‐Ctrl group by repeated measures ANOVA. (I) Protein levels of the NF‐κB/Lcn2 signaling axis in CCI mice pre‐microinjected with AAV‐sh‐Ctrl or AAV‐sh‐*Ntoco* (*n* = 6). ns, not significant, *p < 0.05, **p < 0.01, ***p < 0.001 versus the CCI + AAV‐sh‐Ctrl group.

Neurocognitive tests and MRI analysis were employed to evaluate the effect of *Ntoco* on neurological deficits in vivo. A schematic timeline of the experimental protocol is summarized in Figure 6A. GFP‐conjugated adeno‐associated virus (AAV)‐sh‐*Ntoco* and AAV‐sh‐Ctrl were pre‐injected into the right ventricle of mice before CCI induction (Figure S12A,B). Green fluorescence intensity was observed 28 days post‐injection (Figure S12C). Motor performance was assessed using the rotarod test on days 4–6 following CCI. The latency to fall showed no significant difference among the groups exposed to AAV‐sh‐*Ntoco* or AAV‐sh‐Ctrl (Figure 6B). Spatial memory was evaluated using the MWM on days 8–16 post‐injury. Compared with the CCI + AAV‐sh‐Ctrl group, the escape latency was significantly shortened in the sham‐operated group pre‐injected with AAV‐sh‐Ctrl (days 11–14) or AAV‐sh‐*Ntoco* (days 10, 11, 13, and 14). Notably, CCI mice pre‐injected with AAV‐sh‐*Ntoco* exhibited a decreased latency on days 13 and 14 following CCI compared to those pre‐injected with AAV‐sh‐Ctrl (Figure 6C). Moreover, the frequency of platform III crossings on day 16 post‐injury was significantly higher in the sham+AAV‐sh‐Ctrl, sham+AAV‐sh‐*Ntoco*, and CCI + AAV‐sh‐*Ntoco* groups than that in the CCI + AAV‐sh‐Ctrl group (Figure 6D). The swimming track, swimming speed, and body weight were similar among different groups (Figure 6E,F and Figure S13), suggesting that motor abilities and physical condition did not affect memory performance. MRI scans were conducted on days 0, 3, and 7 after surgery. As shown in Figure 6G,H, CCI + AAV‐sh‐*Ntoco* significantly reduced lesion volume.

We then investigated whether the improved outcomes from AAV‐sh‐*Ntoco* in vivo were caused by activation of the NF‐κB/Lcn2 signaling axis. As shown in Figure 6I and Figure S14, CCI mice pre‐injected with AAV‐sh‐*Ntoco* displayed reduced phosphorylation of IkBα, increased phosphorylation of IKKα/β and p65, and high expression of Hnrnpab and Lcn2. Additionally, we evaluated the safety and toxicity of AAV via HE staining and found no evidence of AAV‐related visceral toxicity or neurotoxicity following injection of the AAV‐sh‐Ctrl virus (Figure S15). Together, these findings indicate that the activation of the NF‐κB/Lcn2 signaling axis by *Ntoco* knockdown contributes to improved brain injury recovery.

## 4 Discussion

**FIGURE 7:**
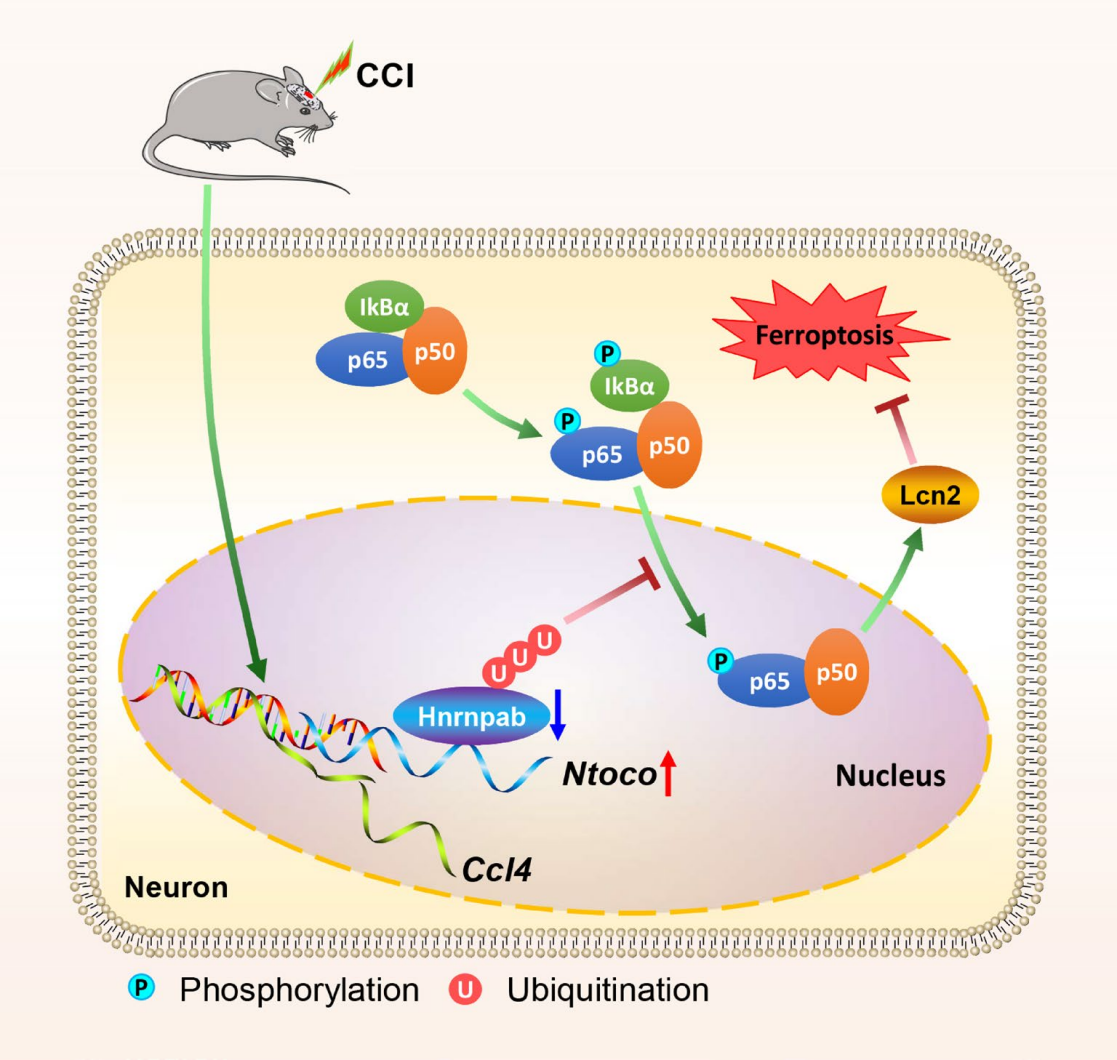
A schematic model for the regulatory role of *Ntoco* in CCI‐induced ferroptosis. *Ntoco* is upregulated following CCI in mice. By interacting with Hnrnpab, *Ntoco* facilitates its ubiquitin‐mediated degradation and dampens the activation of the NF‐κB/Lcn2 signal axis, which eventually triggers ferroptosis.

LncRNAs, functioning as neuromodulators, play vital roles in the pathological processes of TBI [13, 14]. Altered expression levels of lncRNAs after TBI have been reported [23]; however, the mechanism remains elusive. Recent studies have highlighted several lncRNAs, such as *Tubb6*/*Nrf2* [35], *Lgnm* [36], *NEAT1* [37], and *PVT1* [38], in regulating pathways related to TBI and ferroptosis. Unlike these lncRNAs, which primarily target well‐characterized mechanisms, our study identifies *Ntoco* as a novel regulator of ferroptosis in TBI. Specifically, we observed a higher expression of lncRNA *Ntoco* in TBI mice, which plays a pivotal role in neuronal ferroptosis by interacting with Hnrnpab and inducing K48‐linked degradative ubiquitination. Knockdown of *Ntoco* promoted the nuclear translocation of p65, activated the Hnrnpab‐mediated NF‐κB/Lcn2 signal axis, and eventually improved neurological recovery (Figure 7). Taken together, these data implicate that *Ntoco* serves as a key regulator in the development and progression of TBI by modulating ferroptosis.

Ferroptosis, first discovered in 2012, is characterized by intracellular iron accumulation, lipid peroxidation, and abnormal metabolism of glutathione [4, 39]. Growing evidence has revealed that ferroptosis contributes to the pathogenesis of TBI both in vitro and in vivo, and inhibition of ferroptotic cell death exerts a neuroprotective effect against TBI [7, 8, 9, 10, 11]. Ferristatin II, an iron uptake inhibitor, can attenuate TBI‐induced iron homeostatic imbalance and lipid peroxidation, increase MDA levels, and alleviate neuronal injury and neurodegeneration post TBI [40]. Similarly, melatonin and deferoxamine were reported to exert protective effects by suppressing ferroptosis [7, 11, 41]. Mechanistically, melatonin reduces CCI‐induced neurological deficits and cortical lesion volume through metallothionein 2/interleukin‐33/ferritin H signaling [7, 41] and deferoxamine ameliorates neurological dysfunction through the NF‐κB pathway [11]. Our previous work showed that miR‐212‐5p mitigated CCI‐induced ferroptotic neuronal death by targeting *Ptgs2*. In the current study, we demonstrated a nuclear polyadenylated lncRNA, *Ntoco*, which was significantly increased in neurons after CCI. Knockdown of *Ntoco* alleviated RSL3‐induced cytotoxicity and mitochondrial depolarization, decreased intracellular iron accumulation, alleviated spatial memory, and reduced CCI‐induced lesion volume. The evidence strongly supports the potential of ferroptosis‐targeting regulators as therapeutic strategies for CCI.

LncRNAs exert their regulatory mechanism primarily through interaction with RBPs [30, 42]. In this study, we demonstrated the binding of *Ntoco* to Hnrnpab, which is crucial for neural development and neuron cell survival by interacting with a group of mRNAs [43, 44]. Absence of Hnrnpab not only increases sensitivity to excitotoxicity but also results in alternative splicing disorders, loss of dendrites, and learning and memory impairment [43, 45]. Hnrnpab belongs to the heterogeneous nuclear ribonucleoprotein (hnRNP) family, which has multiple roles in translational regulation, pre‐mRNA splicing, and mRNA stabilization [46, 47]. Through interacting with hnRNP I, *lincRNA‐RoR* directly suppresses p53 translation in response to DNA damage [48]. By forming a lncRNA *RP11*/hnRNPA2B1/mRNA complex, the proteasomal degradation of Zeb1 is prevented, leading to its post‐translational upregulation [49]. By interacting with the heat shock protein 90α/HNRNPAB complex, *lnc‐CTSLP4* recruits E3‐ubiquitin ligase ZFP91 and triggers the degradation of HNRNPAB [50]. Similar to these observations, we found that downregulation of *Ntoco* enhanced UPS‐mediated stabilization of Hnrnpab and activated the NF‐κB signaling pathway. These findings indicate a critical role of *Ntoco*‐mediated Hnrnpab ubiquitination and degradation in TBI pathogenesis.

The NF‐κB signaling pathway plays a critical role in regulating ferroptosis [12, 51, 52, 53]. Dimethyl fumarate‐induced ferroptotic cell death relies on inhibiting NF‐κB and JAK/STAT signaling [52]. RSL3‐induced ferroptosis was vulnerable in resistant cells, with activation of NF‐κB, including phosphorylated IKK and IκBα, as well as massive nuclear localization of p65 [53]. Activated NF‐κB signaling resulted in high expression of the iron‐sequestering cytokine Lcn2, which alleviated iron overload‐induced toxicity [12]. These findings indicate that Lcn2 might negatively regulate ferroptosis. Consistent with these results, we observed nuclear translocation of the NF‐κB p65 subunit and activation of the NF‐κB/Lcn2 signaling axis in an Hnrnpab‐dependent manner after *Ntoco* knockdown. Notably, pre‐microinjection of AAV‐mediated *Ntoco* knockdown improved spatial memory and reduced lesion volume in CCI mice. Altogether, we provide evidence that targeting *Ntoco* is a promising therapeutic strategy for CCI management.

This study has several limitations. First, the findings were based on a murine model, and their applicability to human TBI remains to be validated. Second, although we identified the interaction between *Ntoco* and Hnrnpab, other possible pathways and interacting partners were not explored. Third, while the therapeutic potential of targeting *Ntoco* appears promising, additional studies are required to develop effective delivery strategies. Addressing these limitations will provide a more comprehensive understanding of the role of *Ntoco* in TBI and ferroptosis.

In summary, we demonstrated for the first time that *Ntoco* promotes ferroptosis through Hnrnpab‐mediated activation of the NF‐κB/Lcn2 axis in CCI mice. Our study not only reveals a molecular mechanism regulating ferroptosis but also unveils *Ntoco* as a critical regulator in TBI pathology, offering new avenues for therapeutic intervention in TBI.

## Author Contributions

Q.W., H.Z., H.S., X.X., Z.W., T.L., and X.L. performed the experiments; Y.W. and T.W. helped to analyze data; Q.W. drafted the manuscript; L.G. and J.L. conceived and designed the project and revised the manuscript.

## Conflicts of Interest

The authors declare no conflicts of interest.

## Supporting information


Appendix S1

## Data Availability

The datasets generated and/or analyzed during the current study are available in the NCBI Gene Expression Omnibus repository, accessible via https://www.ncbi.nlm.nih.gov/geo/query/acc.cgi?acc=GSE235523. The datasets are available under the terms of the Creative Commons Attribution License (CC BY). Restrictions apply to the availability of some or all data, which were used under license for the current study. Data are available from the authors upon reasonable request and with the permission of the relevant third parties.
