# Supplementary material for: Ntoco Promotes Ferroptosis via Hnrnpab‐Mediated NF‐κB/Lcn2 Axis Following Traumatic Brain Injury in Mice
Source: CNS Neurosci Ther. 2025 Feb 20;31(2):e70282. doi: 10.1111/cns.70282 (PMC11840698; doi:10.1111/cns.70282)
Supplement: Supplementary file 1 — Appendix S1 [file CNS-31-e70282-s001.pdf]

Supplementary Materials

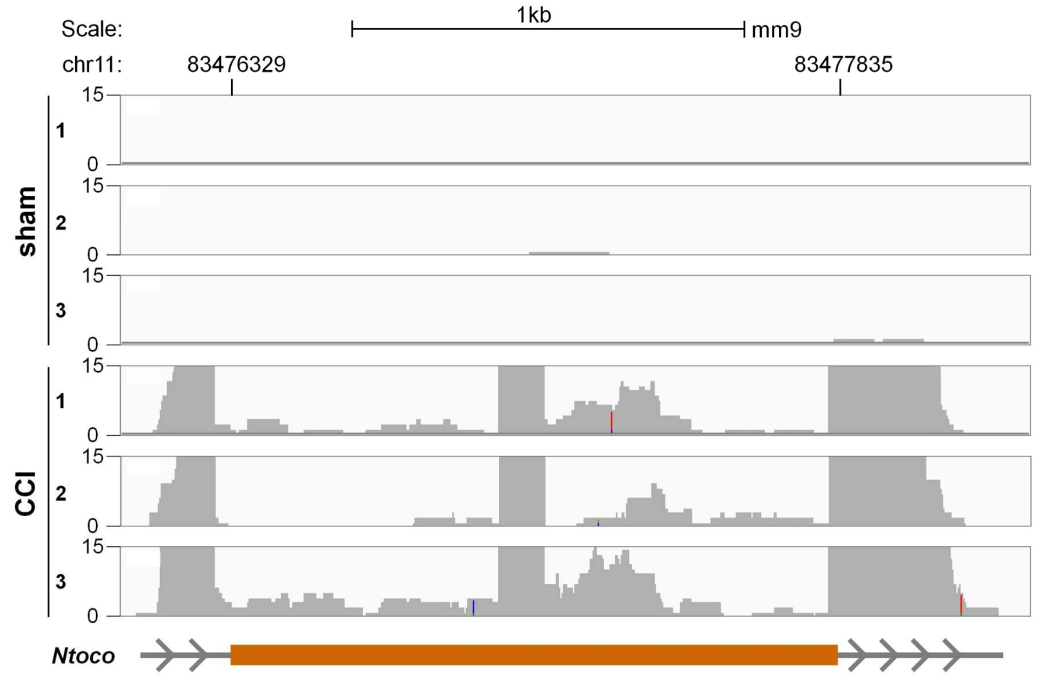

Supplementary Figure 1. Integrative genomics viewer (IGV) visualization of the original reads of *Ntoco* from GSE79441. The stacked reads were increased in the cortex of CCI mice compared with those in the sham group at 24 h post-injury (n = 3).

A

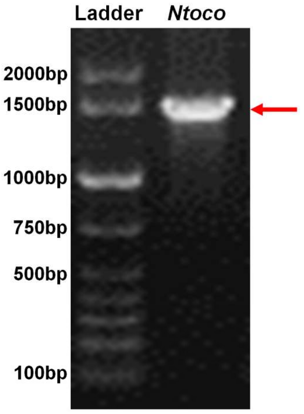

B

>***Ntoco*** (1507nt)

ACCAGAGGGAGCTGGGAAGATTCCCAAGTCTGTTGTTAAACATGGAATCTTCCAGACAGCCAT
TAATCCTTAAGCTCCCAAAAGAGGGAATCCTTCACAGCCAATGGTCAGATATTGAATCAGCTT
TCTCTCTGGTTTAAACTTGTTTGTCTTTATGCCTTGATTTCCCCACCTTTAATATGAAGCAAT
TGAGAGGTGTTCTGAGTTTTGATCTAGTTTCAATCTTCTAGATTTCTTTCCCCTTCTTCTTAG
AGAAGTATCTAGTTAGAGTAGAAATATCATTCTGGGCTGGAGAGATGGCTCAATGGTTAAGAG
CACTGACTGCTCTTCCAGAGGACCTGAGTTCAATTCCCAGCAACCACATGGTGGCTCACAACC
ATCTGTAATAGGATCCGACGCACTCTTCTACTGTGTGTCTGAAGATAGCTGCAGTGCACTCAT
ATAAATAAATAAAAGTTTATTAATCAATTAGTTAATTAATTAAAAAGAAAAAGAAGTCTCATT
CTGGGTGGATGACCATAGATTTCAGGGACCATGTTTTGGGTTCCAGTAGTCCATAAGAATGCT
CAATGAAGATTTATAATGAACAGTGCCTGGTACAGTTTTGACCTTACCCTCACTTTTCCTTCC
AGTGGGCTCTGACCCTCCCACTTCCTGCTGTTTCTCTTACACCTCCCGGCAGCTTCACAGAAG
CTTTGTGATGGATTACTATGAGACCAGCAGTCTTTGCTCCAAGCCAGCTGTGGTGTAAGTACA
CATTGGGATGAGGGCAAGATTCGAAAAGGGCCAGGTTCAGGTGAAAATGTTATCTAACTGACG
GGGGTCAATTCTAAGGGTTGAAGCTCTGCCAAAGAACAGTGAGGTCATGGGGTTCTGGTGGCT
GCTGGATGGGCAGTAAAGTGTGGAGTCAGGAATGGCTTTTGGGGAGGAAGTTGCTACACGCTA
CGGAACTAGAAAACAGAAGATGACGCATACATACTATTTAGATAGATTGCTTAAACTATGTTA
GAAGAGCATGTAGAAAGGGCATTTGAAAAATTGGGAGACATATTCATATTGATTATCAAGCCT
GTTGTTTTCTAATATAGGGAACCCCTGAGGCTGCAGCTACACCTATGGGTCAGAGTTATAGGG
GGTCTAATGTAGGGTCATCCAGAAAGGATGATGTACTATGGCTACCCATACAAACAATGGTCA
AGCAGAACATGATTTCCAAGTCTGCTGTTAAATAGAGTCTATCAGACAGTCATTAGTCCTTAA
GCGTCTGTTTCCTTAAGAATTAACAGCCAATAGAGGGAGATGGTGGACTGGAAGTCAGATATT
GAATGAGCTTTCTCTCTGGTTTAAATGTGTTTGTCTCTATGGCTTGATTTCAAAGGCATAGGC
ATATGTCTTGAGGCAAAGGACAGTGGTAGATGTCCTCTAAGGTCCCATGGGGTTCTCATCTGT
GCTCTCTTTCTTTTACAGATTCCTGACCAAAAGAGGCAGACAGATCTGTGCTAACCCC

Supplementary Figure 2. Full-length sequence of lncRNA *Ntoco*. (A) Agarose gel electrophoresis image of full-length *Ntoco*. (B) Sanger sequencing identification the full-length sequence of *Ntoco* (genome location: chr11:83476329-83477835 (+)).

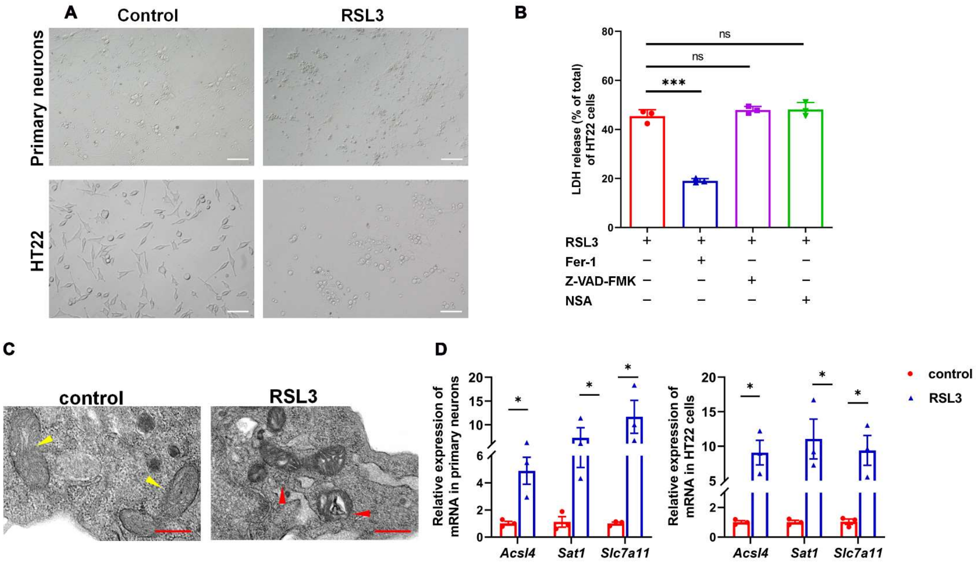

Supplementary Figure 3. Establishment of cellular model of neuronal ferroptosis. (A) Optical microscopy images of primary neurons and HT22 treated with and without RSL3 (3 μM). Scale bar = 20 μm. (B) Lactate dehydrogenase (LDH) cytotoxicity assay showing the LDH release rate in HT22 cells treated with RSL3 and the ferroptosis inhibitor ferrostatin-1 (Fer-1, 0.4 μM), the apoptosis inhibitor zVAD-fmk (10 μM), or the necroptosis inhibitor necrosulfonamide (NSA, 0.5 μM). Data were analyzed using one-way ANOVA with an LSD post hoc test. ns, not significant. *** $P < 0.001$. (C) TEM images of HT22 cells treated with and without RSL3. The yellow arrow indicates normal mitochondria. The red arrow indicates RSL3-induced mitochondrial damage. Scale bar = 500 nm. (D) At 24 h after treatment with RSL3, ferroptosis-related genes were detected using qRT-PCR. * $P < 0.05$ *vs*. the control using Student's *t*-test.

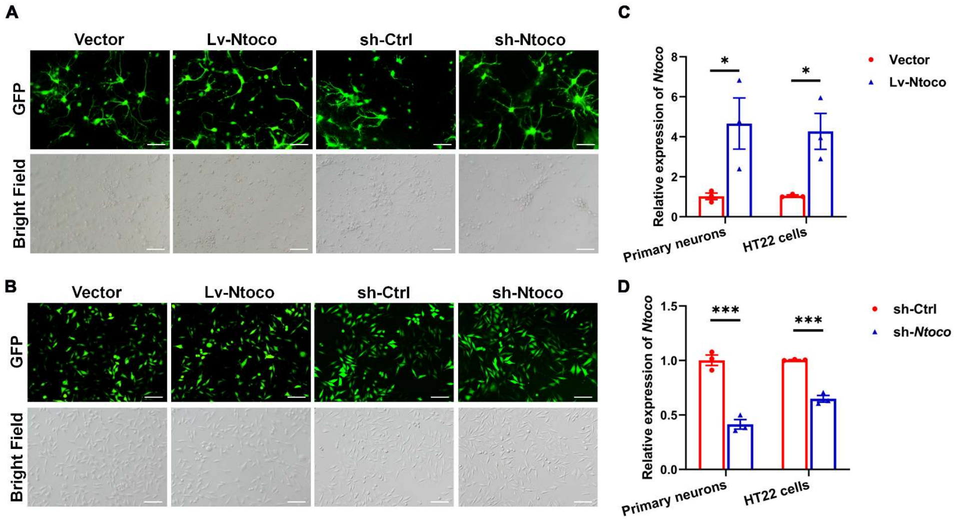

Supplementary Figure 4. Infection efficiency of neurons by lentiviral particles with *Ntoco* overexpression or downregulation. (A-B) Fluorescence microscopy images of primary neurons (scale bar = 10 μm) and HT22 cells (scale bar = 50 μm) infected by lentiviral particles with *Ntoco* overexpression (Lv-*Ntoco*) or downregulation (sh-*Ntoco*). Vector, overexpression control; sh-Ctrl, downregulation control. (C-D) Relative levels of *Ntoco* in primary neurons and HT22 cells were detected using qRT-PCR. * $P < 0.05$ *vs.* the control using Student's *t*-test.

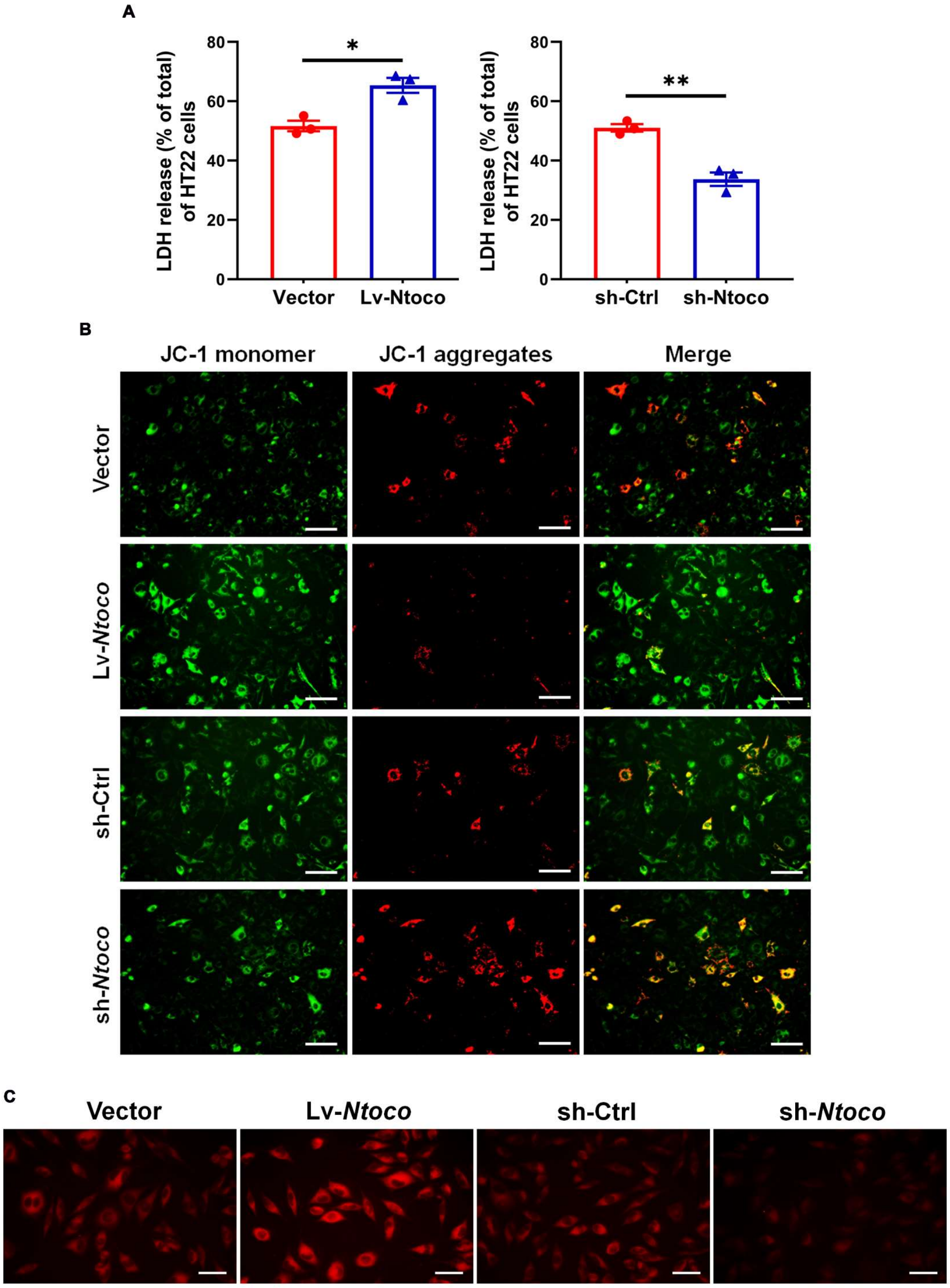

Supplementary Figure 5. *Ntoco* promotes RSL3-induced ferroptosis in HT22 cells. (A-C) HT22 cells with *Ntoco* overexpression (Lv-*Ntoco*) or knockdown (sh-*Ntoco*) were treated with RSL3. After 24 h, the LDH release rate, JC-1, and FerroOrange staining were used to measure RLS3-induced cytotoxicity (A), mitochondrial membrane potential (B) (scale bar = 50 μm), and ferrous ion levels (C) (scale bar = 20 μm), respectively. * $P < 0.05$, ** $P < 0.01$ *vs*. the control using Student's *t*-test.

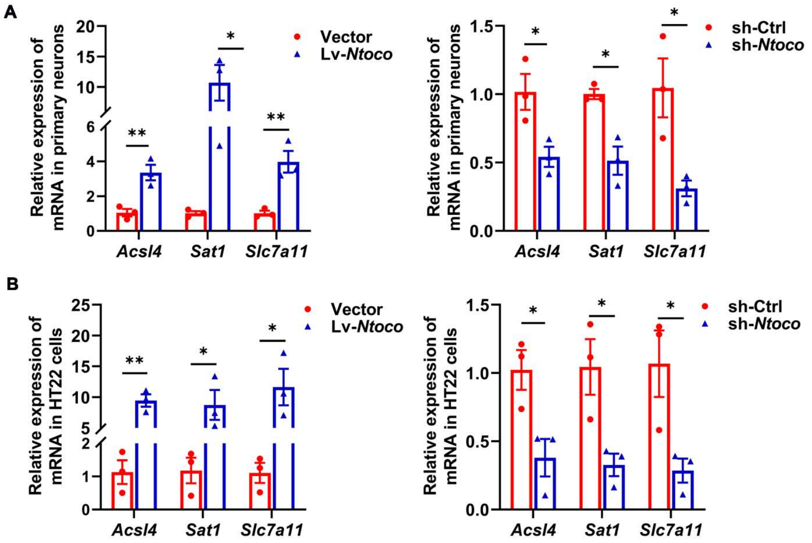

Supplementary Figure 6. *Ntoco* enhances the expression of ferroptosis-related genes. (A-B) In cultured primary neurons and HT22 cells treated with *Ntoco* overexpression (Lv-*Ntoco*) or knockdown (sh-*Ntoco*), the expression of ferroptosis-related genes was examined using qRT-PCR. * $P < 0.05$, **$P < 0.01$ *vs*. the control using Student's *t*-test.

A

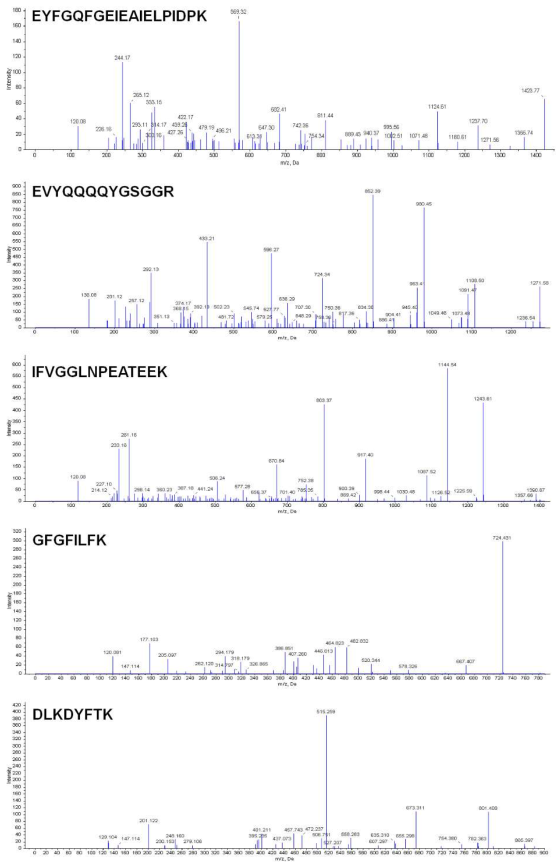

B

Name	Hnrnpab
Length	285aa
Mass	42kDa
Unused	12
Coverage(%)	25.3
Unique PepSeq	EYFGQFGEIEAIELPIDPK,GFGFILFK,IFVGGLNPEATEEK,DLKDYFTK,EVYQQQQYGSGGR
Peptide	5
Unique Peptide	5

Supplementary Figure 7. Hnrnpab is the binding protein of *Ntoco*. (A) Five unique peptides interacting with *Ntoco* were identified by mass spectrometry. (B). Detailed information of *Ntoco* interacting proteins.

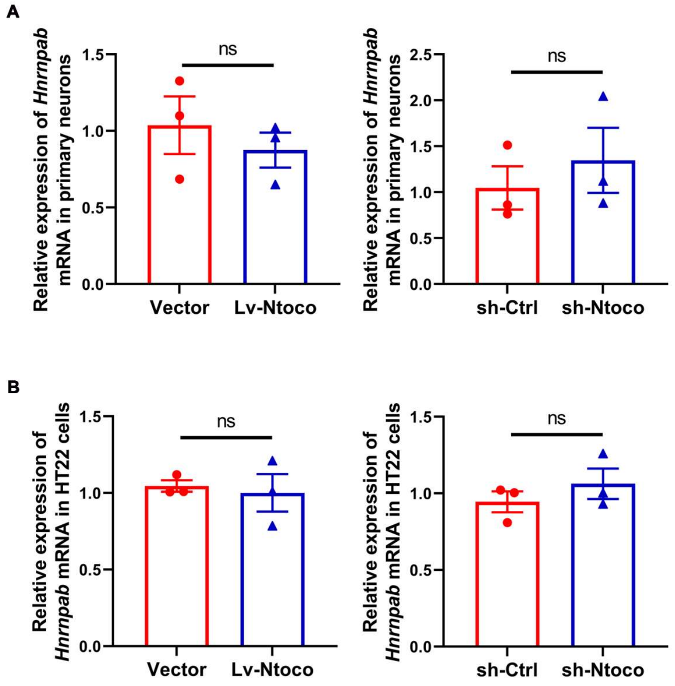

Supplementary Figure 8. *Ntoco* does not influence the transcription of *Hnrnpab*. Levels of *Hnrnpab* mRNA in primary neurons (A) and HT22 cells (B) with either **overexpression** (Lv-*Ntoco*) or knockdown of *Ntoco* (sh-*Ntoco*) were examined using qRT-PCR. Vector, overexpression control. sh-Ctrl, downregulation control. ns, not significant *vs*. the control using Student's *t*-test.

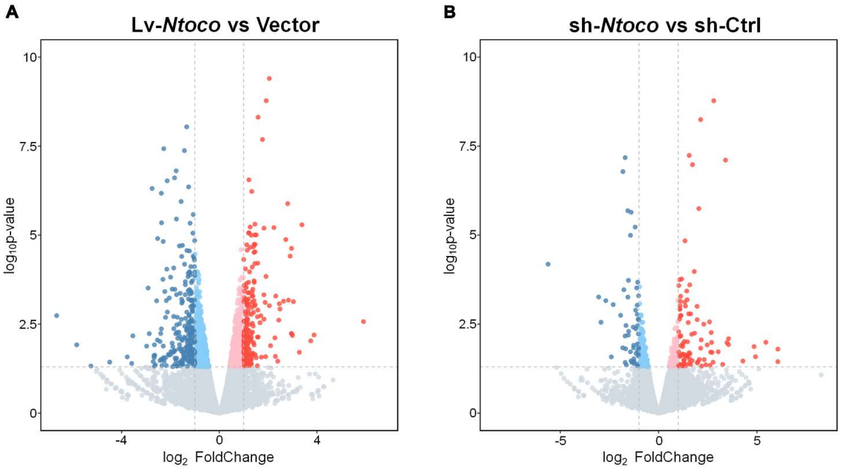

C

Group	KEGG_ID	Pathway	*p*-value
**sh-*Ntoco* vs sh-Ctrl**	mmu04064	NF-kappa B signaling pathway	0.002386
	mmu04668	TNF signaling pathway	0.003282
**Lv-*Ntoco* vs Vector**	mmu04066	HIF-1 signaling pathway	7.88E-06
	mmu04668	TNF signaling pathway	0.000199
	mmu04068	FoxO signaling pathway	0.008714
	mmu04010	MAPK signaling pathway	0.013971
	mmu04392	Hippo signaling pathway - multiple species	0.021385
	mmu04064	NF-kappa B signaling pathway	0.029329
	mmu04014	Ras signaling pathway	0.033269
	mmu04015	Rap1 signaling pathway	0.049191

Supplementary Figure 9. RNA-sequencing analysis identified signal transduction pathways associated with *Ntoco*. (A-B) Volcano plot of gene expression in *Ntoco* overexpressing (Lv-*Ntoco*) and knockdown (sh-*Ntoco*) cells. Red dots indicate upregulated genes, green dots indicate downregulated genes, and gray dots indicate no significant difference compared with the controls. Differentially expressed genes were filtered using $|\log_2 \text{fold change}| > 1.0$ with *P-value* < 0.05. (C) Signal transduction pathways enriched by *Ntoco* overexpression and knockdown.

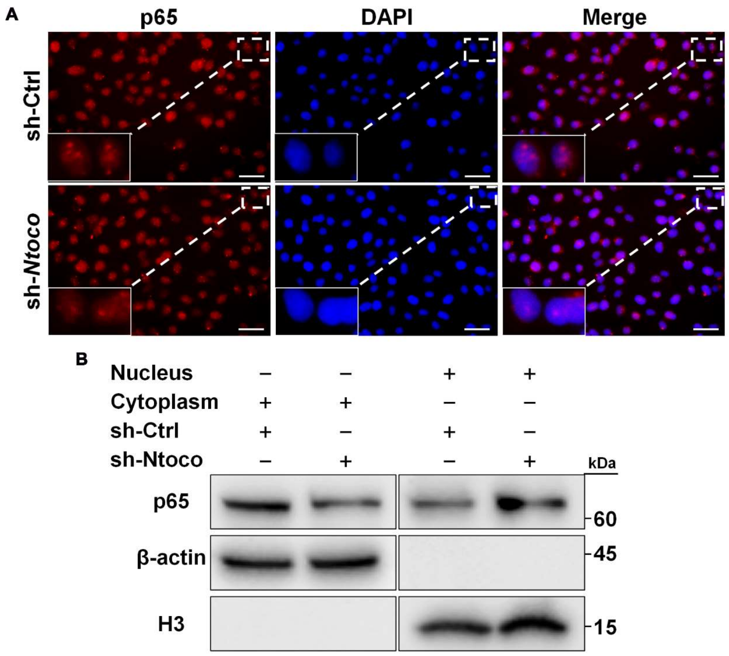

Supplementary Figure 10. Knockdown of *Ntoco* promotes nuclear translocation of the p65 subunit of NF-κB in HT22 cells. (A) Immunofluorescence analysis of the relocalization of p65 in HT22 cells with *Ntoco* knockdown (sh-*Ntoco*). Scale bar: 50 μm. (B) Western blotting revealing the level of the p65 subunit of NF-κB in cytoplasmic and nuclear fractions of sh-*Ntoco* HT22 cells.

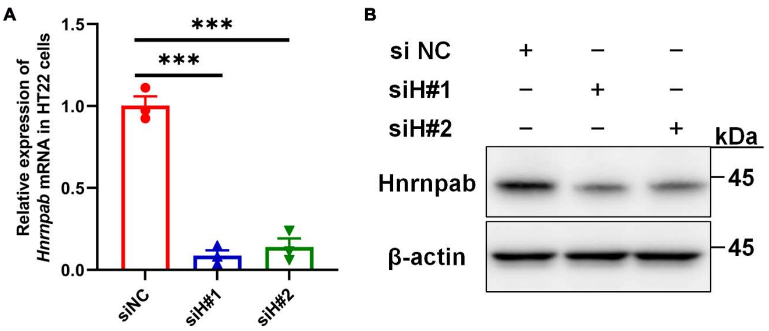

Supplementary Figure 11. Efficiency analysis of *Hnrnpab* knockdown. Two validated small interfering RNAs (siRNAs) against *Hnrnpab* (siH#1 and siH#2) and siRNA control (siNC) were transfected into HT22 cells. At 24 h after transfection, levels of *Hnrnpab* mRNA (A) were detected using qRT-PCR. ***$P < 0.001$ *vs.* the siNC group using one-way ANOVA with an LSD post hoc test; levels of Hnrnpab protein (B) were detected using western blotting.

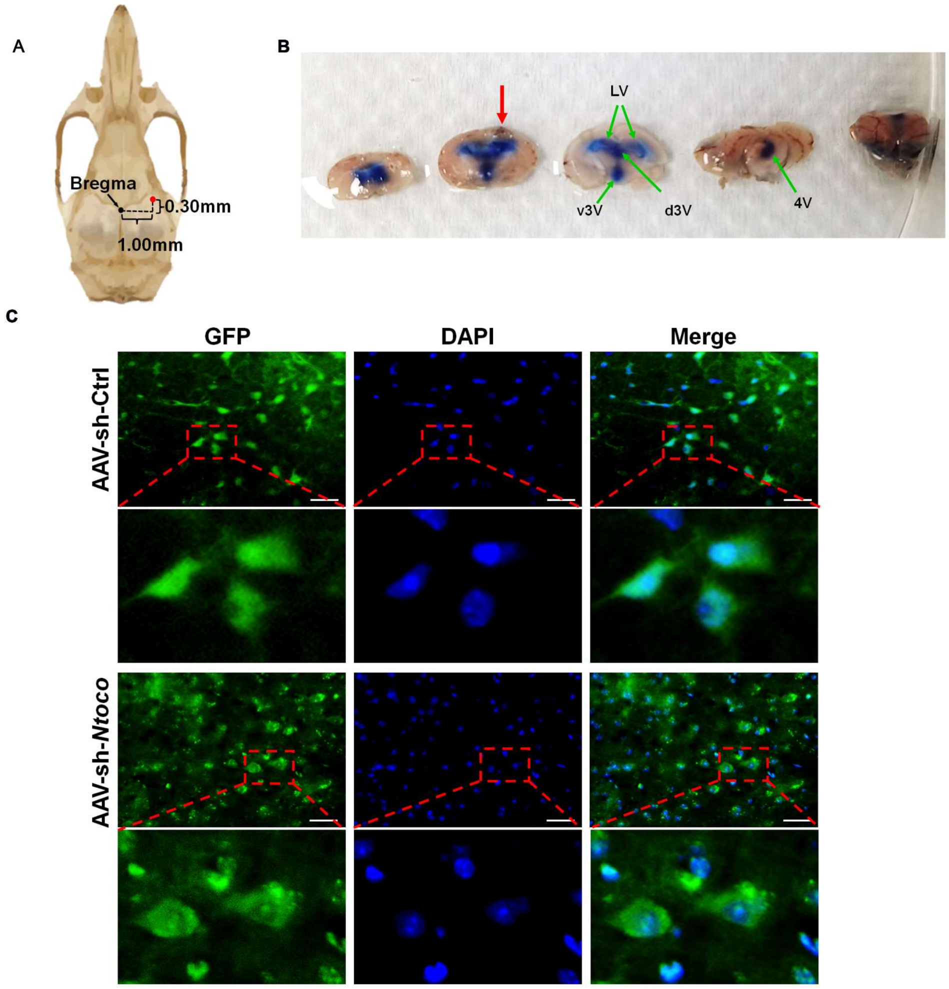

Supplementary Figure 12. AAV microinjection into the lateral ventricle of mice. (A) Patten diagram of the location of AAV microinjection into lateral ventricle (LV). (B) Brain section images of mice after microinjection of bromophenol blue into the lateral ventricle. A red arrow indicates the needle position. V3V, ventral 3rd ventricle; d3V, dorsal 3rd ventricle; 4V, 4th ventricle. (C) Immunofluorescence images of brain sections on 28 days after AAV microinjection. Scale bar: 20 μm.

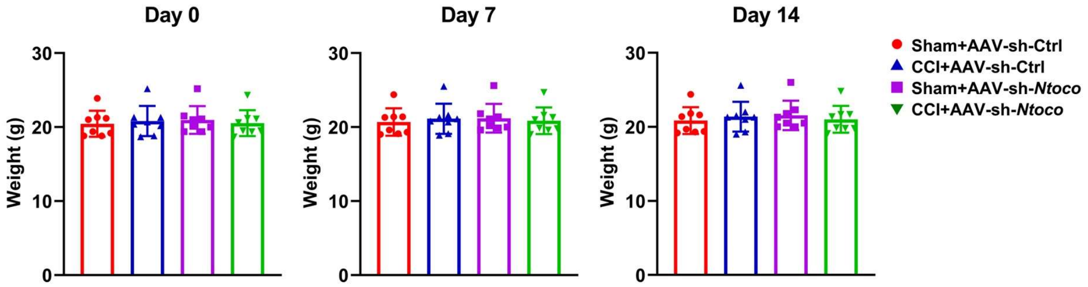

Supplementary Figure 13. AAV microinjection does not influence the body weight of mice. AAV-sh-Ctrl and AAV-sh-*Ntoco* were pre-microinjected into the lateral ventricles 28 days before CCI. On day 0, 7, and 14 post-injuries, the body weight was measured (n = 14).

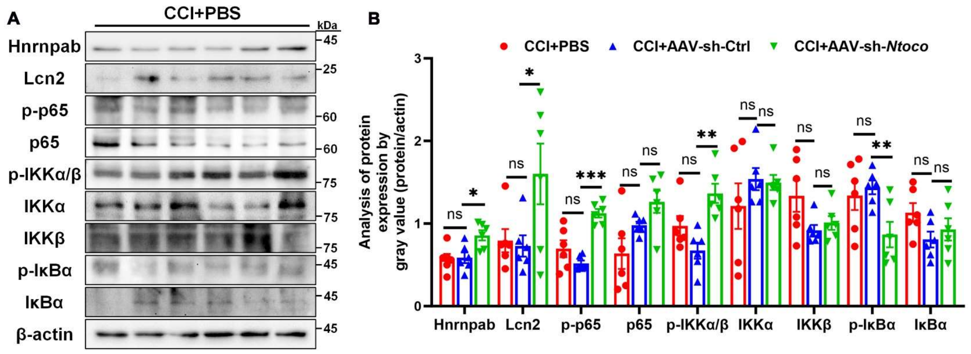

Supplementary Figure 14. Analysis of NF-κB/LCN2 axis-related protein expression levels in response to AAV injection. (A) Protein levels of NF-κB/Lcn2 signaling axis members in CCI mice microinjected with PBS (n=6). (B) Quantification of protein expression levels represented as gray value ratios normalized to β-actin in CCI+PBS, CCI+AAV-sh-Ctrl, and CCI+AAV-sh-*Ntoco* group (n=6). Data were analyzed using one-way ANOVA with the least significant difference (LSD) post hoc test. ns, not significant; * $P < 0.05$, ** $P < 0.01$, *** $P < 0.001$.

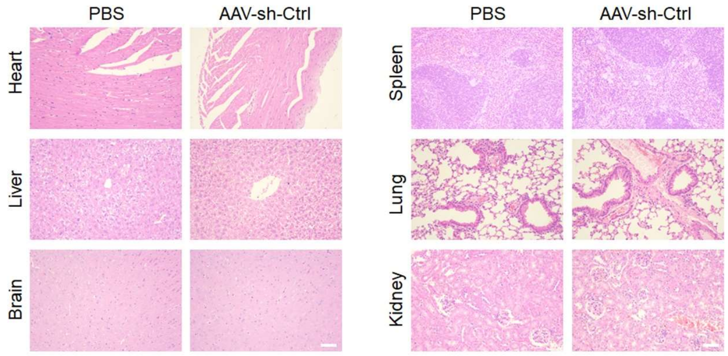

Supplementary Figure 15. Histological Evaluation of Organ Toxicity Following Intracerebroventricular Injection of AAV Virus. Representative H&E-stained histological sections of major organs (heart, liver, brain, spleen, lung, and kidney) from mice injected intracerebroventricularly with PBS (left panels) or AAV-sh-Ctrl (right panels). (Scale bar = 50 μm)

Supplementary Table 1. Primers and siRNAs/shRNAs used in the study

Application	Name	Sequences (5′-3′)
qPCR primers	*Acsl4*-forward	AGCGTTCCTCCAAGTAGACC
	Acsl4-reverse	GTCCTTCGGTCCTAGTCCAG
	Sat1-forward	AAGCCAGGTTGCCATGAAGT
	Sat1-reverse	TTGAAGAGCCTCCATCCCTC
	Slc7a11-forwardr	TCTGACGATGGTGATGCTCT
	Slc7a11-reverse	GGGATGAAGAGAGGCACCTT
	Neat1-forward	GTTCACAGCCCTGCTCAGAT
	Neat1-reverse	GGTTCCAGGCACAATCCTCA
	Malat1-forward	TGCAGTGTGCCAATGTTTCG
	Malat1-reverse	GGCCAGCTGCAAACATTCAA
	GAPDH-forward	TCAGGAGAGTGTTTCCTCGT
	GAPDH-reverse	TGCCGTGAGTGGAGTCATAC
	Ntoco-forward	CCCACTTCCTGCTGTTTCTC
	Ntoco-reverse	CCCCATGACCTCACTGTTCT
	Ccl4-forward	GCCCTCTCTCTCCTCTTGCT
	Ccl4-reverse	GTCTGCCTCTTTTGGTCAGG
	Hnrnpab-forward	AGGCCATTGAGCTTCCAATA
	Hnrnpab-reverse	GGGCAACCTTGATTTCACAC
	IL-6-forward primer	CTGCAAGAGACTTCCATCCAG
	IL-6-reverse primer	AGTGGTATAGACAGGTCTGTTGG
	NOX2-forward primer	TTGGGGCTGAATGTCTTCCT
	NOX2-reverse primer	AGTGCTGACCCAAGGAGTTT
	IL-1β-forward primer	GGAACCCGTGTCTTCCTAAAG
	IL-1β-reverse primer	CTGACTTGGCAGAGGACAAAG
	TNF-α-forward primer	ACCCTCACACTCACAAACCA
	TNF-α-reverse primer	GGCAGAGAGGAGGTTGACTTT
	β-actin-forward	GTGGGAATGGGTCAGAAGGA
	β-actin-reverse	TACATGGCTGGGGTGTTGAA
ChIP primers	*Ntoco*-ChIP-forward	AATCACAGCCACCTGGAAAG
	Ntoco-ChIP-reverse	AATGTTCACACCTGCCACAA
RACE primers	3′ RACE outer primer	TTGCTACACGCTACGGAACT
	3′ RACE inner primer	TAGAGGGAGATGGTGGACTGGA
	5′ RACE RT primer	TGGACTACTGGAACCCAAAACA
	5′ RACE specific primer 1	CACAGTAGAAGAGTGCGTCGGA
	5′ RACE specific primer 2	GTGGGGAAATCAAGGCATAA
	Ntoco full-length PCR forward	ACCAGAGGGAGCTGGGAAGA
	Ntoco full-length PCR reverse	GGGGTTAGCACAGATCTGTCTG
siRNA	siHnrnpab#1-S	CCCAACACUGGACGAUCAATT
	siHnrnpab#1-AS	UUGAUCGUCCAGUGUGGGTT
	siHnrnpab#2-S	GGAAGAUCCUGUGAAGAAATT
	siHnrnpab#2-AS	UUUCUCACAGGAUCUUCCTT
shRNA target	sh-*Ntoco*	GCGTCTGTTTCCTTAAGAAT

Supplementary Table 2. Antibodies used in this study

Name	App./Dilution	Catalog Number	Manufacturer
Anti-Neuron specific beta III Tubulin antibody	WB 1:1000<br>IF 1:200	ab229590	Abcam
Anti-MAP2 antibody	FC 1:100	ab183830	Abcam
Anti-Histone H3 (tri methyl K27) antibody	ChIP 2μg/mL	ab6002	Abcam
Goat Anti-Rabbit IgG	WB 1:10000	ab150077	Abcam
Rabbit IgG	IP 2μg/mL	ab172730	Abcam
Anti-HNRPAB antibody	WB 1:1000	ab199724	Abcam
β-Actin rabbit mAb	WB 1:20000	AC026	Abclonal
Histone H3 rabbit mAb	WB 1:1000	A17562	Abclonal
Anti-Ubiquitin antibody	IP 2μg/mL	ab7254	Abcam
Anti-Ubiquitin (linkage-specific K48) antibody	IP 2μg/mL	ab140601	Abcam
Anti-Ccl4 antibody	WB 1:1000	Ab45690	Abcam
Anti-NF-κB p65 antibody	WB 1:1000<br>IF 1:200	ab16502	Abcam
Lipocalin-2/NGAL rabbit pAb	WB 1:1000	A2092	Abclonal
Anti-NF-κB p65 (phospho S536) antibody	WB 1:1000	ab76302	Abcam
TNF alpha Antibody	WB 1:500	AF7014	Affinity
NOX2 Antibody	WB 1:2000	DF6520	Affinity
IL-1 Beta Polyclonal Antibody	WB 1:2000	bs-0812R	Bioss
IL-6 Rabbit mAb	WB 1:1000	12912	CST
Phospho-IKKα/β (Ser176/180) Rabbit mAb	WB 1:1000	2697	CST
IKKα Antibody	WB 1:1000	2682	CST
IKKβ (D30C6) Rabbit mAb	WB 1:1000	8943	CST
Phospho-IκBα (Ser32/36) (5A5) Mouse mAb	WB 1:1000	9246	CST
IκBα (44D4) Rabbit mAb	WB 1:1000	4812	CST
HRP Goat Anti-Rabbit IgG	WB 1:10000	AS014	Abclonal
Cy3 Goat Anti-Rabbit IgG	IF 1:500	AS007	Abclonal
FITC Goat Anti-Mouse IgG	FC 1:1000	AS001	Abclonal

mAb, monoclonal antibody; pAb, polyclonal antibody; WB, western blotting, IF, immunofluorescence; FC, flow cytometry; IP, immunoprecipitation; ChIP, chromatin immunoprecipitation.
